# Breaking the Summer Dormancy of *Pinellia ternata* by Introducing a Heat Tolerance Receptor-Like Kinase *ERECTA* Gene

**DOI:** 10.3389/fpls.2020.00780

**Published:** 2020-06-24

**Authors:** Seifu Juneidi, Zengyan Gao, Huanran Yin, Nokwanda P. Makunga, Wei Chen, Sheng Hu, Xiaohua Li, Xuebo Hu

**Affiliations:** ^1^Laboratory of Drug Discovery and Molecular Engineering, Department of Medicinal Plants, College of Plant Science and Technology, Huazhong Agricultural University, Wuhan, China; ^2^National-Regional Joint Engineering Research Center in Hubei for Medicinal Plant Breeding and Cultivation, Huazhong Agricultural University, Wuhan, China; ^3^Medicinal Plant Engineering Research Center of Hubei Province, Huazhong Agricultural University, Wuhan, China; ^4^National Key Laboratory of Crop Genetic Improvement and National Center of Plant Gene Research (Wuhan), Huazhong Agricultural University, Wuhan, China; ^5^Department of Botany and Zoology, Stellenbosch University, Stellenbosch, South Africa; ^6^Hubei Cancer Hospital, Wuhan, China

**Keywords:** *ER*, *HMGR*, *Pinellia ternata*, secondary metabolites, summer-dormancy, thermo-tolerance

## Abstract

*Pinellia ternata* is a perennial traditional Chinese medicinal plant that undergoes different phenological patterns of dormancy depending on where it is growing. Plants grown in central and southern China typically display two growth cycles every year before and after hot summer days, exhibiting a summer dormancy. However, germplasms from these areas do not go into a dormancy phase in northern China where the summer monthly average temperatures range from 29–31°C. The northern China herbal growers prefer plant stocks from central China due to their longer growing quality and better tuber harvests. Here, we introduced a heat responsive *receptor-like kinase ERECTA* (*ER*) gene into *P. ternata* to explore changes in the growth cycle which were aimed at disrupting the summer dormancy. The *3-hydroxy-3-methylglutaryl coenzyme A reductase* (*HMGR*) gene was also co-transformed with *ER* to improve the commercial trait. For the thermo-tolerance evaluation, all plants were treated with high temperatures (35°C/40°C) in a growth chamber or grown in natural field temperature in an isolated field before measurement of different agricultural, biochemical and physiological traits. The transgenics showed significantly (*P* < 0.05) higher heat tolerance, maintaining healthy vegetative growth unlike the empty vector (*EV*) harboring controls that became chlorotic and necrotic. Better performance in some of the monitored physiological traits was evident for overexpression lines exposed to the heat stress. In open isolated field trials, the transgenic genotypes did not show a summer dormancy but had a survival rate of 84–95%. The tuber biomasses were also significantly (*P* < 0.05) higher for the transgenic lines as compared to the *EV* controls, except for line *ER118*. Metabolites analysis indicated that the *HMGR* overexpressing lines (*HMGR* or*HMGR* + *ER*) exhibited significantly higher amounts of bioactive compounds including aromadendrene-4, 10-diol and 4, 8, 13-cyclotetradecatriene-1, 3-diol, 1, 5, 9-trimethyl-12-(1-methylethyl). Our findings show that the summer dormancy of *P. ternata* which is a naturally evolved trait, can be removed by a single heat responsive gene. The study contributes to generating heat tolerant new *Pinellia* varieties with enhanced commercially valuable chemicals.

## Introduction

*Pinellia ternata* (Thunb) Breit (Araceae) is a perennial plant that normally grows in humid and shady environments with moderate temperatures (Hu, 1989). It is native to Eastern Asian countries, namely; Korea, Japan and China. In China, *Pinellia* is cultivated mainly in the central and southwest provinces (Zhang et al., 2013) because it is an important medicinal plant. Some plants have evolved a dormancy mechanism during winter or summer as an adaptation to cope with drought or heat stresses (Gillespie and Volaire, 2017). *P. ternata* is naturally susceptible to high temperatures in the summer season and its aerial parts start to wither and senesce from mid-July when it slows down its normal vegetative growth. In central and southern China, where the summer temperatures can reach above 35°C, the plant undergoes seasonal dormancy. However, in northern China, where the summer temperatures are relatively lower than in central China, the *Pinellia* plant grows continuously without showing a seasonal summer dormancy. Herbal growers generally prefer genotypes of this species that originate from central China because they have a better yield (Ma et al., 2006; Zhang et al., 2013). Strategies that alter the phenology of these genotypes, which circumvent the heat response that induces summer dormancy, are thus highly desirable.

Recently, much progress has also been made in improving plant tolerance to heat stress through genetic modifications. One of these strategies includes overexpressing the *ERECTA* gene of the family Leucine-Rich Repeat Receptor-Like Protein Kinases (LRR-RLKs) into Arabidopsis and economically important crops. Members of the *LRR-RLK* gene family have been shown to improve the ability of plants to tolerate heat when plants are challenged with high temperature stress for a long duration (Shpak et al., 2004; Shen et al., 2015). In Arabidopsis, the *ER* gene family is composed of *ERECTA*, *ERECTA-LIKE1* and *ERECTA-LIKE2*. These genes are known to regulate multiple aspects of plant development including proximodistal axis elongation, anther differentiation, integument growth promotion, xylem radial expansion and vascular bundle differentiation (Shpak, 2013).

*P. ternata* is one of the most popular herbal medicines that used for the treatment of insomnia, tumor, eclampsia, obesity and depression for hundreds of years in Chinese Traditional Medicine (Iwasa et al., 2014). Its tubers are the main source of bioactive phytochemicals that show antitussive, antioxidant, antibacterial, anti-inflammatory and antiemetic actions (Hongying et al., 2015). Several specialized metabolites have been identified in this species including terpenoids, lignanoids, alkaloids, and phenylpropanoids (Wu et al., 2011; Iwasa et al., 2014). Some of these accumulate at low levels (Zuo et al., 2012; Lee et al., 2016) and the application of transgenic technologies can offer solutions to generate *Pinellia* cultivar(s) with improved chemical, agricultural and/or commercial traits.

In spite of this, genetic modifications in this species are few. As example, Tang et al. (2008) introduced the aroA-M12 glyphosate resistance gene and Jin et al. (2009) generated fungal resistant *P. ternata* expressing the chitinase gene (ech42) and β-1, 3-glucanase gene (gluc78). Zhu et al. (2018) transformed with the sHSP genes that encode for small heat shock proteins. However, none of these studies directly focused on specialized metabolism. To our knowledge there are no reports available with regard to genetic modifications of the *HMGR* gene, a rate limiting enzymes, to improve the production of specialized metabolites of *P. ternata*. The *HMGR* gene encodes an enzyme that is crucial in the biosynthesis of terpenoids and alkaloids (Bansal et al., 2018). We, thus, hypothesized that the overexpression of *HMGR* could enhance the secondary metabolite content and provide new evidence for its regulatory function in heat stress tolerance.

**FIGURE 1:**
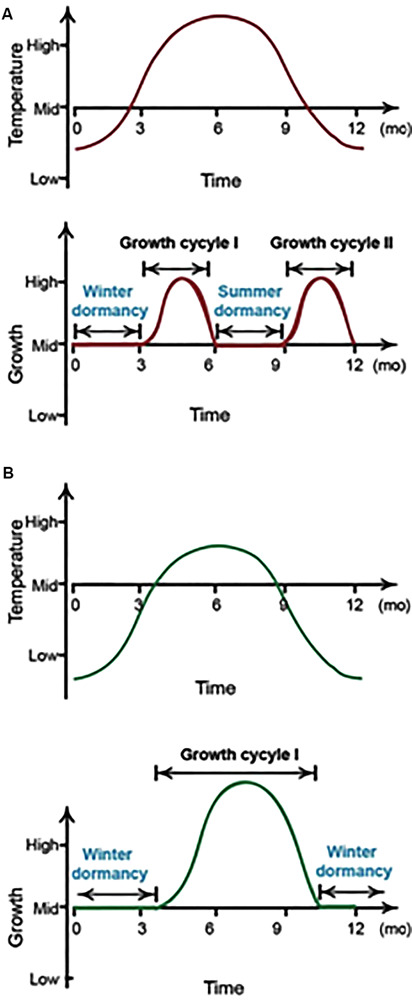
A depiction of *P. ternata* growth cycles. In southern and central China, *P. ternata* develops two seasonal dormancy periods every year **(A)**, whereas it has one dormancy period per year in northern China **(B)**. mo, month.

The aims of this study were two fold. Initially, we explored how a heterologous Arabidopsis *ER* gene would change the basic life cycle of *P. ternata* as our target was to break the heat-inducible summer dormancy pattern (Figure 1). The study also examined the role of the *HMGR* gene in regulating heat tolerance in *P. ternata*. In other taxa, the HMGR enzyme responds to various biotic and abiotic stresses, leading to altered metabolite profiles. To the best of our knowledge, this is the first report to break plant summer dormancy by overexpressing a heat responsive gene concomitant to improving the chemo-type of *Pinellia* via *HMGR* up-regulation of specialized metabolism.

## Materials and Methods

### Construction of Expression Vectors

The cDNA clone of *ER* in *pCAMBIA1301* expression vector was obtained from Professor He Zuhua, Shanghai Institute of Life Sciences (Chinese Academy of Sciences, China). The heat stress response of the *ER* gene was previously described by Shen et al. (2015). The plasmid was extracted using a Kang Ji Century extraction kit before it was analyzed using 1% (w/v) agarose gel electrophoresis. A nanodrop2000 spectrophotometer was used throughout the study to quantify nucleic acids (Thermo Fisher Scientific, United States).

The *PESC-HIS-mvaA* vector harboring the *HMGR* gene was a generous gift from Professor Zhang Yansheng [Wuhan Botanical Garden (Chinese Academy of Sciences, China)]. The gene was excised from the vector using restriction digestion with *Nco*l and *Bst*EII restriction enzymes before 10 μg of the digestion was applied to a 1% (w/v) agarose/ethidium bromide gel. The cut gel fragment was purified using Kanto’s PCR gel clean up kit. Two different constructs were made i.e., *CaMV35:HMGR* or *CaMV35:HMGR* + *CaMV35:ER*. These were inserted into the *pCAMBIA1301-ER* vector, containing the *ER* gene. A gene construct, the *CaMV35S:HMGR* + *CaMV35S:ER* was generated using a ligation reaction with a ratio of 3:1 ratio (v/v) regarding the *HMGR* fragments to *pCAMBIA1301-ER* vector. The gene construct was transformed into DH5α competent cells using a heat shock method (Froger and Hall, 2007). The transformants were then grown on Luria Bertoni selective medium (50 mg L^–1^ kanamycin), and the positive clones were screened using a colony PCR. The correct recombinant orientation of the insert to the vector was verified by sequencing. Five hundred nano gram of plasmid DNA was used as a template for sequencing, and a sequencing comparison was made electronically via BLAST of National Centre for Biotechnology Information (NCBI).

### *Agrobacterium* Transformation

The *CaMV35:HMGR* or *CaMV35:HMGR* + *CaMV35:ER* constructs were cloned into the *pCAMBIA1301* expression vector, and then transformed into *Agrobacterium* via electroporation (Yang et al., 2000). The transformants were diluted in 1 ml antibiotic-free yeast extract peptone (YEP) medium and incubated for 1 h. The cells were recovered by centrifugation at 3,500 *g* for 5 min. The collected cells were then grown on a YEP selective medium containing 50 mg L^–1^ rifampicin and 50 mg L^–1^ kanamycin for 2 days at 28°C. Colony PCR was used to verify positive clones.

### Explant Preparation, Growth Condition and Plant Transformation

Healthy *P. ternata* tubers were collected from the Dong Yanni Pinellia co-operatives production site (30°24′N 112°54′E), Qianjiang, Hubei, China. The plant species was identified by Professor Hu Xuebo, Department of Medicinal Plants, Huazhong Agricultural University (HZAU), China. The surfaces of tubers were disinfected with 75% (v/v) ethanol for 10 min, followed by soaking in 0.1% HgCl_2_ (w/v) for 20 min. Decontaminated tubers were then grown on Murashige and Skoog (1962) basal medium to generate *in vitro* stock plants. The plant growth chamber conditions were set at 25 ± 2°C temperature, 3,000 Lux light intensity, 16/8 h light/dark photoperiod and ∼65%relative humidity. Leaf discs (1–1.5 cm), excised from 5-week-old plantlets, were placed together with an *Agrobacterium* suspension culture, harboring the transgenes, for 15 min. After infection, the plant material was transferred to co-cultivation MS medium containing 100 μg L^–1^ acetosyringone and kept in dark area for 72 h at 25°C as previously described by Xu et al. (2005). Three days later, the plant material was thoroughly washed with sterile water, blotted dry with Whatman No.1 filter papers and transferred to MS solid medium to generate transgenic plants. Shoot regeneration was induced on a selection MS medium containing 30 g L^–1^ sucrose, 1 mg L^–1^ Kinetin (KT), 30 mg L^–1^ hygromycin, 50 mg L^–1^ kanamycin and 50 mg L^–1^ rifampicin. After 5 weeks, the shoots were transferred to a root inducing MS medium supplemented with 30 g L^–1^ sucrose, 1 mg L^–1^ KT, 0.1 mg L^–1^ 2, 4-dichlorophenoxyacetic acid (2, 4-D), 0.1 mg L^–1^1-naphthalane acetic acid (NAA), 30 mg L^–1^ hygromycin, 50 mg L^–1^ kanamycin and 50 mg L^–1^ rifampicin. The positive transformants were then verified by using PCR.

For each genetic construct (*CaMV35:HMGR*, *CaMV35:ER* or *CaMV35:HMGR* + *CaMV35:ER*), twenty individual transgenic plant lines were produced. Ten transgenic lines of each construct that looked morphologically similar to untransformed plants were selected. Transgene expression levels were also used for selection of transgenic lines using RT-PCR (see description in the next section). A preliminarily heat resistance screening test, where, transgenic plants were subjected to a 40°C heat treatment for 7 days was also used. Apart from higher temperature, all other conditions in the growth chamber were kept same, as previously mentioned. The best three thermo-tolerant lines of each construct were selected for further studies. The empty vector (hereafter denoted as *EV*) harboring plants were used as controls.

### Real-Time PCR

Total RNA was isolated from leaves of 10-week-old *Pinellia* plantlets using CTAB-pBIOZOL reagents according to the manufacturer’s instructions (BIOER). The RNA was treated with an RNase-free DNase before cDNA was generated from 1 μg of total RNA using a SuperReal PreMix Plus (SYBR Green) TIANGEN kit. Two microliters of cDNA were used for qPCR analysis with SYBR Green PCR master mix using the aqTOWER 2.2 real- time PCR detection systems (Analytikjena, Jene, Germany). The total volume of the reaction was adjusted to 20 μL. The primer sequences used are in Supplementary Table S1. Three biological replicates were used for gene expression analysis. The 2^–Δ^ ^Δ^ ^*Ct*^ method (Livak, 2001) was used to quantify the relative expression levels of *HMGR, ER* and *HMGR* + *ER* (hereafter denoted as *H* + *E*). The *Actin* gene was used as a housekeeping control.

### Heat Treatment Response of *ER* Overexpressing *Pinellia*

#### Plant Growth and Morphological Characterization

The selected three transgenic lines of each *ER* or *H* + *E*, and the *EV* control plantlets were grown on MS medium in a growth chamber for 8 weeks as mentioned earlier. Plantlets were transplanted into soil and grown in a growth culture room that had similar growth conditions with the growth chamber. After 2 weeks, the seedlings were subjected to different temperatures, namely high temperature treatment (40°C, 15-day) or a long-term warm temperature regime (35°C, 90-day) plus natural field temperature in a pot trial in an isolated field. High and long-term temperature treatments were adopted from the method of Shen et al. (2015) with slight modifications. Briefly, 10-week-old seedlings were grown at 40°C (heat stress) for 15-day followed by a 15-day recovery growth period at 25°C (normal growth temperature)-to determine high temperature stress survival and recovering rates. Likewise, 10-week-old seedlings were grown at 35°C for 90-day for a prolonged warm temperature stress survival rate assessment. The heat survival rate was defined as the ratio of the number of plants maintaining green shoots to the total number of plants that were subjected to the stress temperatures. The ability to survive these temperature regimes was recorded daily. Changes in plant morphology were monitored and recorded daily. Plants were watered manually every other day to maintain soil moisture during the heat treatments. The rate of recovery was also monitored for the plants that were treated with a high temperature at 40°C. The recovery rate was described as the ratio of the number of plants that exhibited normal development when growing at the normal growth temperature at 25 ± 2°C.

### Physiological Response Measurements

*In situ* leaf chlorophyll content was measured before and during heat stress treatments at 0, 24, 48, and 72 h using a SPAD 502 plus chlorophyll meter (Konica Minolta, Japan). Briefly, 5–10 fully expanded intact, clean and healthy leaves were selected and measured for relative *in situ* chlorophyll content. A single leaf was measured at least five times and an average of the measurements was considered as the representative of leaf chlorophyll content. The net photosynthetic rate, transpiration rate, internal CO_2_ concentration and stomatal conductance of 10-week-old plantlets were measured using a LI-COR6400 (LI-COR Biosciences, United States) gas exchange system according to Shen et al. (2015). A batch of 15–25 plants was measured before (0 h) and after a 48 h heat treatment using three biological replicates. The instantaneous water-use efficiency was determined from the ratio of the net photosynthetic rate to transpiration rate, and the relative water content was measured according to Xu et al. (2014).

### Oxidative Stress Damage Analysis

Different measurements linked to ion leakage, H_2_O_2_ and O_2_^–^ detection, lipid peroxidation, total protein content and superoxide dismutase (SOD) activity were recorded. Leaves of 10-week-old *Pinellia* were used for the detection of ion leakage (Zang et al., 2017). Total protein content was measured spectrophotometrically using Bradford protein assay method (Krohn, 2001). The H_2_O_2_ tests (Alexieva et al., 2001) and leaf lipid peroxidation malonaldehyde (MDA) assay (Shiu et al., 2004) was conducted. The activity of SOD was measured similarly to Beauchamp and Fridovich (1971). The histochemical staining of detached leaves that were infiltrated with 3, 3′-diaminobenzidin (DAB) or nitrotetrazolium blue chloride (NBT)were evaluated for the accumulation of H_2_O_2_ or superoxide radical anions (O_2_^–^), respectively (Kumar et al., 2014).

### Open Field Trials

Field tests were conducted in 2018 and 2019 at the HZAU experimental station, Wuhan, China. The *ER* overexpressing plantlets were grown on MS medium for 8 weeks before being transferred to soil and acclimatized *ex vitro* for 2 weeks in a growth culture room. In each experiment the plants were grown in pots (9 cm height × 10.16 cm width) containing a soil mix of perlite: humus (1:1). Ten-week-old *Pinellia* microplants were then transplanted to an experimental site during the spring (April–June) and the summer (July–September) seasons for an open natural field experiment. Daily records of relative humidity and temperatures (low, high, and average) were obtained from the Wuhan meteorological service station (Supplementary Table S4). Thirty to forty plants of each individual line in a randomized designed were grown and analyzed for different agronomic traits including plant height, root length, total plant biomass, survival rates, and tuber biomass. The agronomic traits records were collected in spring (April–June) and summer (June–September) seasons for a seasonal comparison in terms of temperature differences.

### Extraction of Metabolites

Each tuber was cut into sections and then dried at room temperature, for 2 days, before the material was ground to a fine powder using a pestle and mortar. The powder was extracted in methanol (1:5 ratio; w/v) for 1 h. The homogenate was then sonicated at 60°C for 110 min using an ultra-sonicator bath (Rico Scientific Industries, India). The extract was filtered using Whatman No. 1 and cellulose membrane filter paper and afterward the organic solvent was evaporated to dryness using a RV 3 V-C rotary evaporator (IKA, United States). Each residue was dissolved in 5 ml methanol before gas chromatography mass spectrometry (GC-MS).

### Analysis of Metabolites by GC-MS

The GC-MS analysis was conducted using a Shimadzu GC-MS-TQ8040 system (Kyoto, Japan) coupled directly to a MS detector. The chromatographic separation was performed using the Rtx-5MS (5% phenyl-95% polydimethyl siloxane; 30 m × 0.25 mm ID, 0.25 μm) fused-silica column supplied by Restek (Bellefonte, United States). Helium was used as carrier gas at a constant flow rate of 1 L min^–1^. The ion source and interface temperatures were set at 230 and 290°C, respectively. Sample injection used a split mode (ratio 10:1) and the injector port temperature was set to 280°C whilst the GC oven temperature program was set as follows: 80°C (3 min hold), ramped to 110°C (5 min hold) at 10°C min^–1^, increased to 190°C (3 min hold), ramped to 220°C (4 min hold) at 10°C min^–1^, and then increased to 280°C (13 min hold) at 15°C min^–1^. The MS detector, operated at 70 eV, was set to scan from 60 to 665 atomic mass units. The compounds were identified by comparing spectra of the analyzed samples with the National Institute of Standards and Technology (NIST) library, 2008 and Wiley Registry of Mass Spectral Data, 8th Edition. High match scores (>95%) were considered for compound identification (Lee et al., 2016) and where possible this was checked with published literature.

### Statistical Analysis

One-way ANOVA was used for data analysis, and the significant differences between the control and the transgenic plants were using a Fisher’s Least Significant Difference (LSD) test (*P* < 0.05). Applications were conducted with the SPSS statistical program (version 23).

## Results

### Gene Expression and Transgenic Plant Screening

**FIGURE 2:**
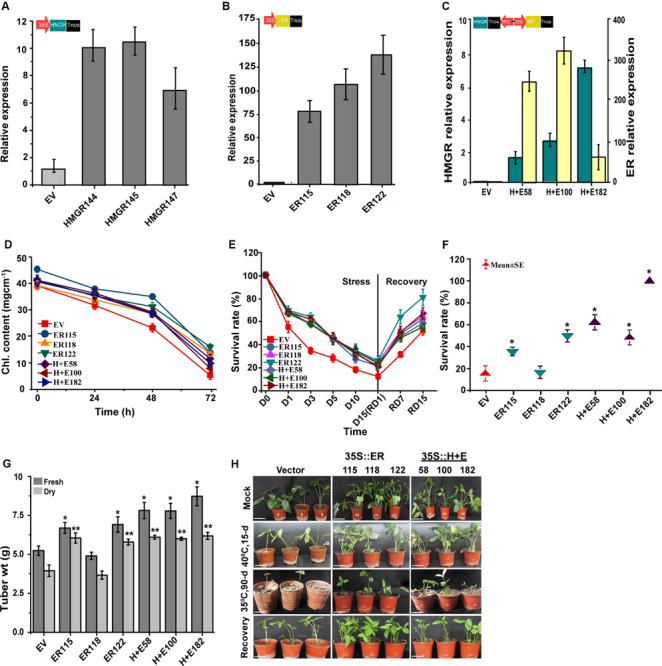
ER enhances thermo-tolerance of *P. ternata*. The relative expression levels of **(A)** HMGR, **(B)** ER and **(C)** H + E in 10-week-old *P. ternate* overexpressing *HMGR, ER* or *H* + *E* under the control of *CaMV35S* promoter. The *Actin* gene was used as a control to normalize internal expression level. **(D)** SPAD leaf chlorophyll content of the empty vector, *ER* and *H* + *E* genotypes sampled at 0, 24, 48, and 72 h. **(E)** Heat stress survival rate of the empty vector, *ER* and *H* + *E* genotypes subjected to a high temperature (40°C, 15-day) and their recovery at a normal growth temperature (25 ± 2°C, 15-day). **(F)** Long-term warm temperature survival rate (35°C, 90-day). **(G)** Tuber weights of the transgenic *Pinellia* plants grown in a growth chamber at 35°C, 90-day and recovered under normal growth temperature (25 ± 2°C, 9 months), 9–12 plants each. **(H)** Phenotype of 10-week-old transgenic *P. terneta* grown at 25 ± 2°C (the first upper panels) and subsequently grown for 15-day at 40°C (the second middle panels), followed by 15-day growth recovery at 25 ± 2°C (the fourth lowest panels) and plants grown for 90-day at 35°C (the third middle panels). Scale bar 1 cm. *P* < 0.05, by one-way ANOVA, LSD for comparisons; Chl., chlorophyll; D, day; RD, recovery day.

The *ER*, *HMGR* and *HMGR* + *ER* genes were constitutively overexpressed in *P. ternata* under the transcriptional control of *CaMV35S* promoter to gain insight into their functions. In total, 20 transgenic lines of each genotype were produced. The transcript levels were significantly (*P* < 0.05) increased in all transgenic plants and hardly detected in the *EV* controls (Supplementary Figure S1). To test the functionality of these genes, the transgenic plants were subjected to high temperature (40°C, 7-day) as part of preliminary screening for thermo-tolerance. Together with the qRT-PCR analysis, three best heat tolerant lines of each group (*ER*: 115,118,122; *H* + *E*: 58,100,182) were selected (Figures 2A–C) and used to continue the study. Under heat stress, the *HMGR* transgenic lines were similar in their morphological appearance to the control plants with the empty vector (refer Supplementary Tables S2, S3 for recorded phenotypic traits).

### Overexpression of *ER* Confers Thermo-Tolerance of *P. ternata*

Temperature stress suppressed the growth of both the transgenic *ER* overexpression (*ER*-OE) and the *EV* controls when plants were exposed to a temperature of 40°C for 15 days. However, the stress effect was more severe in the *EV* controls than the transgenic genotypes (Figures 2D–H). Under normal growth temperatures, there were no obvious differences in plant growth, morphology or biomass as compared to the *EV* controls. But, under heat stress, a different scenario was noticed where the *EV* controls rapidly wilted, showing signs of leaf necrosis and chlorosis. The *EV* controls also exhibited stunted growth and distortion of leaf shape, reduced leaf size and drying of leaf veins whereas the leaves of the transgenic lines remained relatively green and healthy with minor apparent morphological deformities.

We measured chlorophyll content of the intact leaves to test the effect of *ER*-OE in alleviating high temperature stress and/or its possible role in reducing chlorophyll damage. The plantlets regenerated initially at 25 ± 2°C were transferred to the high temperature treatment of 40°C, and the leaves were sampled at different time points (0, 24, 48, and 72 h) for *in situ* chlorophyll content. No differences were observed between the *EV* controls and the transgenic plants when plants were grown at 25 ± 2°C. The chlorophyll content ranged from 42.1 ± 1.3 to 46.5 ± 0.9 mg cm^–1^. The chlorophyll content was significantly lowered when plants were exposed to heat stress irrespective of their transgenic status but the effect was more pronounced for the *EV* controls at each sampled time point. After 72 h, the highest and the lowest chlorophyll contents were measured for the *ER122* (24.6 ± 0.8 mg cm^–1^) and the *EV* controls (16.5 ± 1.2 mg cm^–1^), respectively (Figure 2D).

Under heat stress, the stress tolerance of all the genotypes showed a decline over the 15-day of treatment and this result were statistically significant. Despite this, the heat stress resistance was remarkably higher for the transgenic genotypes than the *EV* controls at each tested time point. On the 15th day, the highest tolerance was recorded for the *ER122* line (26.5%) followed by the *ER118* (24.8%) and *H* + *E100* (23.7%) transgenic genotypes. As expected the *EV* controls had the lowest tolerance as only 12.4% of *EV* controls survived high temperature treatment on day 15. The rate of recovery can indicate how detrimental elevated temperatures are to plants and for the transgenic genotypes, the recovery rate was significantly higher at each sampled time point versus the *EV* controls. Out of the transgenic lines, the *ER122* and the *EV* genotypes had the best revival rates of 80.7% and the lowest 52% at termination of the experiment, respectively. During the course of the prolonged moderate heat treatment (35°C, 90-day), the leaves of the *EV* controls turned to a yellow, wilted and eventually died. On the other hand, the leaves of the transgenic lines remained green; sustaining their growth and development much better as compared to the *EV* controls (Figures 2E,F,H).

### *ER* Overexpression Alters the Physiological Responses of *P. ternata* Against Heat Stress

**FIGURE 3:**
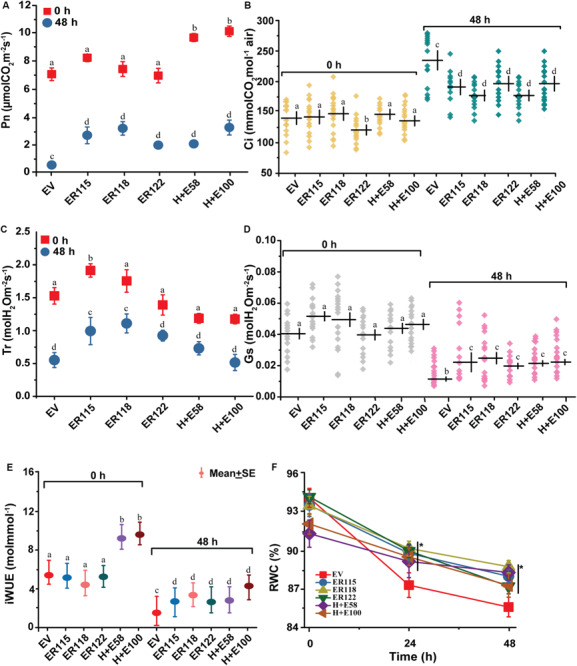
Changes in gas exchange system and water using efficiency. The empty vector control plants and the transgenic plants were grown at normal growth temperature (25 ± 2°C) or under heat stress (40°C) condition. **(A)** Pn, net photosynthetic rate; **(B)** Tr, rate of transpiration; **(C)** Ci, intercellular CO_2_ content **(D)** Gs, stomatal conductance and **(E)** iWUE, instantaneous water use efficiency. Plants were sampled before (0 h) and after (48 h) heat stress treatment. **(F)** RWC, Relative water content. The leaves were sampled at 0, 24, and 48 h time points. Each point represents the mean ± SE of five measurements of 20–25 individual plants. The *P* value was calculated using one-way ANOVA and are indicated by asterisk (*) or different letters on error bars when treatments significantly differ from the controls (*P* < 0.05).

To assess the physiological responses of *ER*-OE *P. ternata*, the net photosynthetic rate, transpiration rate, internal CO_2_ concentration, stomatal conductance, instantaneous water-use efficiency and relative water content were measured before (0 h) and after (48 h) the heat stress treatment (Figures 3A–F). Except for the *H* + *E58* and *H* + *E100* lines, no differences in net photosynthetic rate measurements were observed between the transgenic and the *EV* controls at 25 ± 2°C. With elevated temperature of 40°C, differences were observed between the transgenic and the *EV* controls. Photosynthetic rates were significantly higher in the transgenic lines as compared to the *EV* controls (Figure 3A). Apart from the *ER115* (under normal and stress conditions) and the *ER118* lines (under the heat stress condition), there was no significant difference in transpiration rate between the transgenic and the *EV* controls (Figure 3B). With regards to the intercellular CO_2_ content, the differences between the transgenic and the *EV* controls were not significant at the normal growth temperature, except for the *ER122* line. The internal CO_2_ concentration content was significantly increased when plants were exposed to different heat stressing regimes in all genotypes but the variation was substantial for the *EV* controls (Figure 3C).

The instantaneous water-use efficiency describes the ratio of the net photosynthetic rate to transpiration rate, and changes in either of the two components causes variation in instantaneous water-use efficiency of a plant. Essentially, both parameters are often driven by the plant’s stomatal conductance. For this reason, we measured the stomatal conductance before and after heat treatments. The *ER-*OE genotypes were characterized by much higher leaf stomatal conductance than the *EV* controls with exposure to high heat (Figure 3D). The transgenic lines exhibited significantly higher instantaneous water-use efficiency than the *EV* controls under the heat treatment (Figure 3E). The data shows that the transgenic plants maintained a significantly higher rate of carbon assimilation (2.75–4.13 folds) as compared to the *EV* controls when under heat stress. Leaf rolling became a morphological feature that was associated with plants under heat stress and this was more prominent the longer the period of stress. As a result, we were unable to properly measure the physiological responses of plants from this point onward. The relative water content of the excised plant leaves, which were measured over the time points of 0, 24, and 48 h, were statistically insignificant under normal growth temperatures. After 24 h of heat exposure, however, the relative water content of the *EV* controls declined more rapidly from 94.8 to 87.1%. Values of 10.4% for the *EV* controls and 4.1–7.2% for the transgenic lines were recorded at the end of the 48 h heat stress treatment (Figure 3F).

### *ER* Reduces Oxidative Stress Damages

**FIGURE 4:**
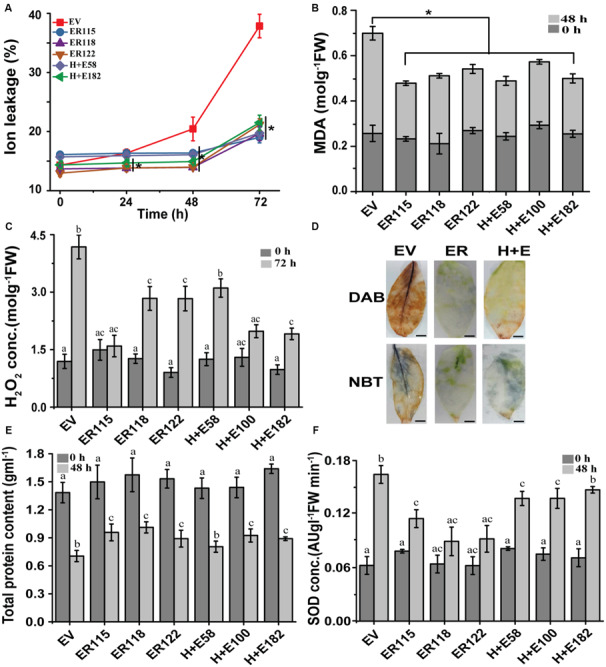
Heat stress-induced reactive oxygen species and oxidative damage analysis. **(A)** Cellular ion leakage over the time course of 72 h. The plants were sampled at 0, 24, 48, and 72 h. **(B)** Malonaldehyde (MDA) leaf content before (0 h) and after 72 h heat treatment. **(C)** H_2_O_2_ content pre- and post-heat treatment. **(D)** H_2_O_2_ and O_2_^–^ levels detected in 10-week-old leaves stained with Diaminobenzidine (DAB) and Nitrotetrazolium blue chloride (NBT), respectively. The assessment was performed before (0 h) and after (48 h) heat treatment (40°C). A brown polymerization product is due to H_2_O_2_ accumulation and a blue coloration is because of O_2_^–^ level, Scale, 1 cm. **(E)** Total protein content, and **(F)** SOD activity before (0 h) and after (48 h) heat treatment. The plants were grown at 25 ± 2°C (normal growth temperature) or 40°C (under temperature stress).

To better understand the role of the *ER* gene at a cellular level with regards to thermos-tolerance, various biochemical assays were carried out during the course of this study (Figure 4). No obvious differences in ion leakage were observed at normal growth temperatures between the transgenic genotypes and the *EV* controls. The electrolyte efflux started increasing at 24 h post-treatment in the *EV* controls. The ion efflux increased by 1.15, 1.5 and 2.7 fold at 24, 48, and 72 h in the *EV* controls, respectively. On the other hand, the transgenic plants showed only slight changes (1.1–1.7 fold) at the end of 72 h (Figure 4A). Similarly, no apparent differences were observed in lipid peroxidation and H_2_O_2_ efflux between untreated transgenic and the *EV* controls. High temperatures caused a rise in lipid peroxidation and rates of 18% in the *EV* controls and 0.086% (*H* + *E58*) to 8% (*ER118*) in the transgenic genotypes (Figure 4B). After 72 h, the level of H_2_O_2_ was higher in *EV* controls as compared to the transgenic genotypes (Figure 4C). After 48 h of the heat treatment, DAB and NBT leaf stains were more pronounced in the *EV* controls than the transgenic lines, indicating higher accumulations of H_2_O_2_ and O_2_^–^, respectively (Figure 4D). Furthermore, the total protein content was significantly reduced in the *EV* controls as result of the 48 h long heat stress as compared to the transgenics (Figure 4E). Superoxide dismutase activity was noticeable in the *EV* controls after the 48 h heat stress treatment as expected. Of interest, we could not measure any marked differences between plants grown under normal and the heat stress condition for the *ER118* and *ER122* lines.

### Field Trial Plant Growth Performance

**FIGURE 5:**
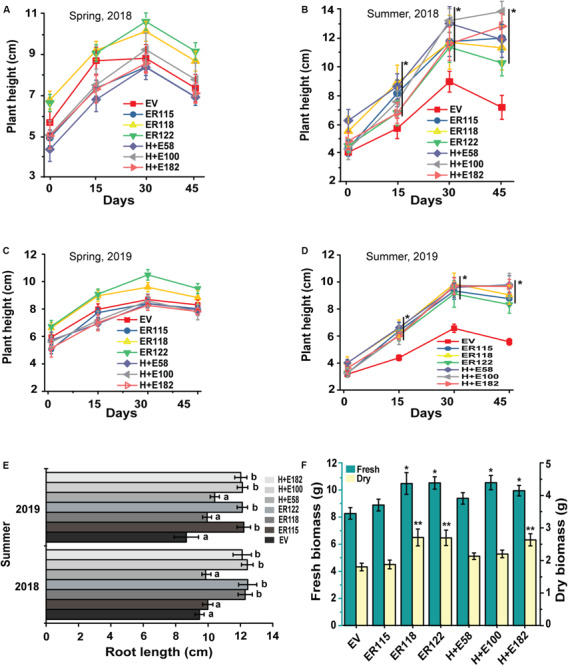
ER improves the growth of *P. ternata* seedlings during the summer heat wave in open natural field. Plant height: **(A)** Spring-2018, **(B)** summer-2018, **(C)** spring-2019 and **(D)** summer-2019, **(E)** Root length, **(F)** Seedling fresh and dry biomass. The root length and plant biomass were recorded after 45-days. The *P* value was calculated using one-way ANOVA and are indicated by asterisk (*) or different letters on error bars when the treatments are significantly different from the empty vector controls (*P* < 0.05).

The *ER* overexpressing *Pinellia* plants showed a promising heat tolerance under fully controlled growth chamber conditions. To validate this observation, the plantlets were transferred to an open natural field and monitored for field growth performances. Meanwhile, plant growth was monitored also as part of a long-term heat treatment (35°C, 90-day) (Figures 2F–H). The daily maximum temperatures range from 16 to 29°C in spring and 29–37°C in summer. In spring, no obvious growth differences were noticed between the transgenic and the *EV* controls (Figures 5A,C and Supplementary Table S5), but notable traits differences were observed in the summer season between the transgenic genotypes and the *EV* controls (Figures 5B,D–F). During the hottest days in summer, the transgenics grew faster than the *EV* controls. The *EV* controls were unable to sustain their normal growth and development and their leaves became smaller. We also noticed more indicators of stress such as leaf rolling, drying and abscission (Supplementary Figure S3). Poor root development for the *EV* controls was obvious whereas the transgenics maintained normal growth and development, generating high biomass values.

**FIGURE 6:**
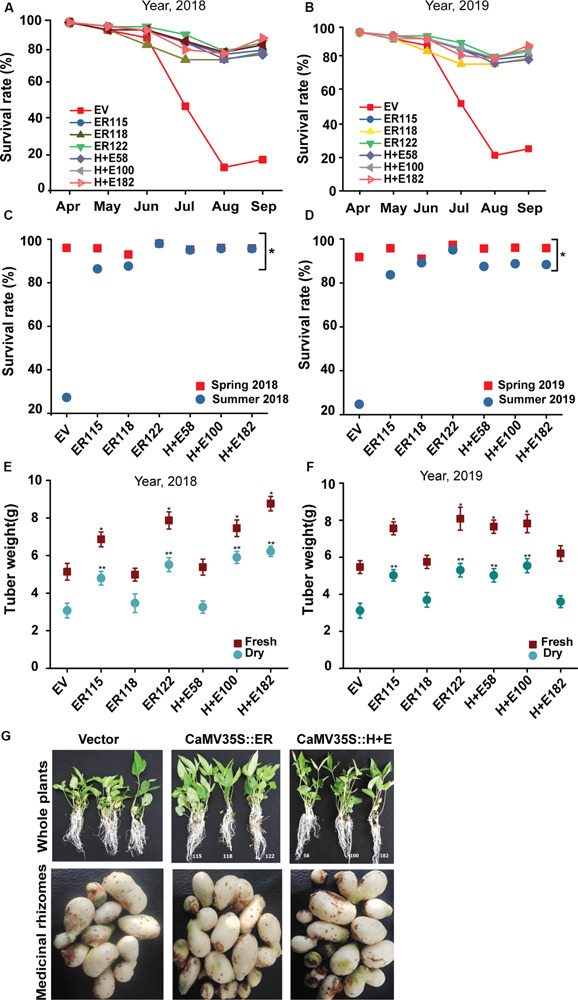
ER breaks the summer dormancy and improves tuber biomass. The plant survival rates were monitored in year **(A)** 2018 and **(B)** 2019. The proportions of survived plants were also computed at the end of spring and summer seasons **(C)** 2018 and **(D)** 2019. Analysis of tuber weight in **(E)** 2018 and **(F)** 2019, 25–30 plants each. The tuber weights were recorded per pot. **(G)** Phenotype of 16-week-old *P. terneta* seedlings that grown the last 6 weeks in open natural field (the first upper panels) and tuber morphology harvested at the end of summer (the second lower panels), scale 1 cm.

The results indicated that no remarkable survival rate differences were observed between the transgenic and the *EV* controls in spring as almost all the transgenic and the *EV* controls survived well (Figures 6A–D). However, the survival rate of the *EV* group was drastically reduced during summer. It is important to point out that the data we obtained did not show any significant growth differences among the transgenic genotypes both in the summer and during spring seasons. The results revealed that both the fresh and the dry tuber weighs were significantly (*P* < 0.05) higher for the transgenic genotypes as compared to the *EV* controls. Only the line *ER118* was similar in its growth measurements to the *EV* controls both in the growth chamber and in open isolated field trials.

### *HMGR* Overexpression Changes the Metabolites Content of *P. ternata*

**FIGURE 7:**
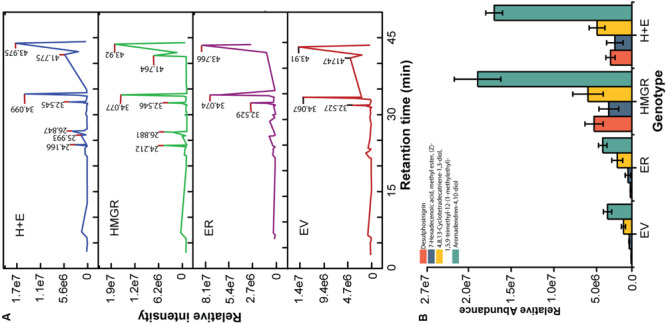
HMGR enhances the metabolites content of *P. ternata*. The methanolic tuber extracts were used for metabolite analysis. **(A)** Total ion chromatograms and **(B)** the relative abundance of differently detected compounds in the transgenic lines. The intensity of peak is the mean of three *HMGR* overexpressing genotypes or the mean of three control plants.

**FIGURE 8:**
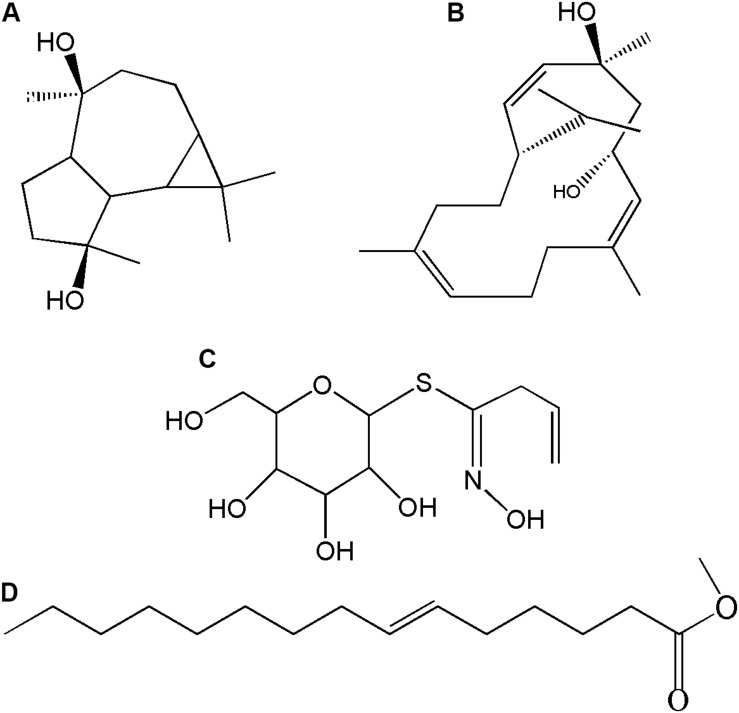
Chemical structure of differently produced compounds in the *HMGR* overexpressing transgenic *P. ternata* tuber extracts. **(A)** Aromadendrene-4,10-diol; **(B)** 4,8,13-Cyclotetradecatriene- 1,3-diol, 1,5,9-trimethyl-12-(1-methylethyl)-; **(C)** Desulphosinigrin and **(D)** 7-Hexadecenoic acid, methyl ester, (Z)-.

The *HMGR* is a key gene in the biosynthesis of terpenoids. Twenty transgenic plants of each genetic construct and qRT-PCR gene expression analysis confirmed the transgenic transformation status of regenerants (Figures 2A,C). The preliminary screening and selection data are indicated in the Supplementary Material (Supplementary Figure S2). The *EV* and the *ER* plants were used as controls in assessing the roles of *HMGR-*OE in altering the metabolite contents. The relative abundances of the differently detected compounds are shown in Supplementary Table S6. Generally, similar compounds were detected in both the controls and the transgenic plants, but some of the compounds occurred at higher concentrations in some of the lines such as aromadendrene-4, 10-diol; 4, 8, 13-cyclotetradecatriene-1, 3-diol, 1, 5, 9-trimethyl-12-(1-methylethyl)-; desulphosinigrin and 7-hexadecenoic acid, methyl ester. The first two compounds belong to terpenoids, whereas the latter two compounds are an alkaloid and a fatty acid ester, respectively. These compounds were differently produced in the *HMGR* transgenic genotypes (Figure 7B). Figure 8 shows the chemical structure of compounds that were differently detected in the transgenic lines.

## Discussion

### *ER* Overexpression Removes the Summer Dormancy of *P. ternata*

Heat stress has detrimental impacts on plant growth, development, and yield worldwide (Long and Ort, 2010). Plants have evolved various mechanisms to cope with various stressing conditions (Kotak et al., 2007; Shen et al., 2015). *P. ternata* adapts to a summer dormancy status as part of its natural regime to cope with climatic conditions when growing in hot environments. To break the summer dormancy of *P. ternata*, the *ER* gene was successfully overexpressed, producing a batch of transgenic lines (Figures 2B,C and Supplementary Figures S1B–D). The *ER* gene has been characterized as an important heat and drought stress regulator in plants. It controls plant growth from the early stages, throughout the developmental processes to the maturity of the plants in Arabidopsis (Qu et al., 2017), tomato and rice (Shen et al., 2015) and poplar (Xing et al., 2011). Similarly, *ER-*OE *P. ternata* lines showed a significantly improved plant growth performance, heat stress survival and recovery rates as compared to the *EV* controls in this study.

It was hypothesized that *ER-*OE could enhance *P. ternata* thermo-tolerance, allowing the plant to cope under such conditions (Figures 1A,B). From a plant physiological viewpoint, we have shown that the transgenic genotypes had markedly higher chlorophyll content (Figure 2D) and better carbon assimilating capacity (Figure 3A) as compared to the *EV* controls. The CaMV35S:ER and CaMV35S:HMGR + ER leaves were greener and healthier than the *EV* controls confirming normal photosynthesis in the transgenic genotypes. Temperature stress affects the physiological and biochemical response mechanisms of a plant (Djanaguiraman et al., 2010; Hasanuzzaman et al., 2013). Although the net photosynthetic rate and chlorophyll concentrations decreased during the heat treatment, these effects were more pronounced in the *EV* controls than the transgenic lines. This possibly indicates the extent of chlorophyll degradation and/or oxidative damage of the Photosystem II (PSII) in the *EV* controls. Heat stress primarily causes injury to the chlorophyll pigments and eventually leads to inhibition of the photosystems and enzyme activities (Hasanuzzaman et al., 2013). Higher net photosynthetic rates were recorded for the *H* + *E58* and *H* + *E100* lines and transpiration rate for the *ER115* and *ER118* genotypes at normal growth temperature. Such observed minor differences among transgenic lines might be attributed to the somaclonal variations that are due to *in vitro* culture or pre-existed in the explants. The gas exchange data suggest that carbon fixation was more efficient in the transgenic lines than the *EV* controls during heat stress (Figures 3C,D). Such data are consistent with other reports (Hector et al., 2012; Shen et al., 2015).

Since *ER* has been characterized as a major player in regulating transpiration efficiency (Masle et al., 2005), many studies have demonstrated its role in enhancing instantaneous water-use efficiency under different environmental stresses (Linzhou et al., 2013; Shen et al., 2015). It is interesting to note that the instantaneous water-use efficiency was, however, enhanced in all the transgenic genotypes, reflecting their higher carbon fixation efficiency (Figure 3E). The relative water content was also significantly higher for the transgenic genotypes than the *EV* controls (Figure 3F). The ability of plants to manage their water content effectively and control their transpiration rate is regarded as a good indicator for heat stress resistance when they are under stress (Xu et al., 2014).

Cell injury associated with plasma membrane disruptions and/or cell wall ruptures are the typical plant cell heat stress indicators, and electrolyte leakage, lipid peroxidation and/or ROS accumulation are exacerbated as a result of stress in plants (Djanaguiraman et al., 2010; Song et al., 2012; Shen et al., 2015). The *ER*-OE transgenic lines were better able to tolerate heat stress as shown by the lower levels of biochemical stress indicators (Figures 4A–D). The accumulation of H_2_O_2_ and O_2_^–^ was greater in the *EV* controls possibly leading to effects that impaired their growth. The *ERECTA* is thought to maintain the integrity of the plasma membrane during heat stress, and also has a role in scavenging the ROS that affect the structural integrity of the plasma membrane. There is still much that is unknown with regards to the cellular and molecular heat stress tolerance mechanisms linked to *ER* regulation. Stress responses in plants are highly complex and SOD is involved in the antioxidant defense system, and hence the elevated levels in those plants that are more susceptible to ROS production during heat stress. The lower SOD activity in the transgenics suggested better thermal tolerance as opposed to the *EV* controls that had much higher levels.

Field trials were important in validating the experimental differences that were observed under controlled growth conditions. In the present study, the *ER-*OE *P. ternata* had a better heat stress endurance and growth performance during field trials. The *ER* transgenic plants exhibited distinctive phenotypes as compared to the *EV* controls as they grew more rapidly and had longer roots. Biomass production is also another important growth parameter that is often severely affected when plants grow under stress environments (Figures 5A–F) and those that are able to cope with the stress show better growth performance and this boosted biomass is often linked to rate of carbon assimilation (Linzhou et al., 2013; Shen et al., 2015). In this study, 84%-95% of the transgenic *Pinellia* plants survived the summer heat wave, exhibiting a higher tuber biomass as compared to the *EV* controls with a survival rate of 25% (Figures 6E–G). This effect may not necessarily be unique to *Pinellia* as in a previous study of Shen et al. (2015), *ER* overexpressed in tomato and rice led to better adaption to the high summer temperature in field tests at Wuhan and Shanghai. In Mediterranean climates, the summer dormancy that is associated with plants endemic to the region is said to be an ecological advantage that confers higher levels of survival under severe drought stress (Balachowski et al., 2016) and the water content is possibly a determining factor that regulates summer dormancy (Shane et al., 2010). In central and southern China, where *P. ternata* shows summer dormancy, drought is less likely to be an evolutionary selection factor as Wuhan, as an example, receives average precipitation that exceeds 1,315 mm per year. It is thus more likely that temperature might be the major driving force controlling the summer dormancy of *P. ternata*. Our findings suggest that a single heat tolerance heterologous gene can break the summer dormancy patterns of this species. Little has been done to understand the underlying mechanisms that control summer dormancy and practices to break dormancy in plants appear to species and cultivar specific. In this study we show that heat-stress imposed dormancy is negated in *ER-*OE *P. ternata* (Figure 4) and this is a new mechanism that has practical applications.

### *HMGR* Alters the Metabolite Content of *P. ternata*

The *HMGR* has been studied as a key and rate-limiting enzyme in the biosynthesis of a diverse spectrum of specialized metabolites in plants, and some examples include *A. thaliana* and *Panax ginseng* (Kim et al., 2014), *Salvia miltiorrhiza* (Dai et al., 2011) and *Gossypium hirsutem* (Loguercio et al., 1999). It provides a suite of precursor molecules that ultimately lead to synthesis of a wide range of terpenoids (Dai et al., 2011; Bansal et al., 2018). For instance, the *HMGR* overexpression or down-regulation leads to the apparent increase/decrease of the down-stream tanshinone and squalene content in transgenic *Salvia* roots (Dai et al., 2011). In the present study, *P. ternata*, overexpressing *HMGR* enhanced the content of aromadendrene-4, 10-diol (sesqueterpene) and 4, 8, 13-cyclotetradecatriene-1, 3-diol, 1, 5, 9-trimethyl-12-(1-methylethyl) (diterpene) in transgenic plants. This finding is in line with the work of others that conclude *HMGR*-OE improves the content of sesquiterpenes and diterpenes in plants (Tholl, 2006; Dai et al., 2011; Kim et al., 2014). These compounds have been previously reported from *P. ternata* plant (Wang et al., 1995; Fu, 2005; Iwasa et al., 2014). Two non-terpenoid compounds that were identified as desulphosinigrin and 7-hexadecenoic acid methyl ester (Zhang et al., 2002; Wang et al., 2008) were also enhanced in transgenic genotypes.

Up-regulation of the rate-limiting enzymes are used to enhance the secondary metabolite production in plants and these metabolites are well known in assisting plants with coping with abiotic and biotic factors (Mahmoud and Croteau, 2002; Tholl, 2006; Dai et al., 2011). In our current study, however, the *HMGR*-OE transgenic plants had similar growth and development phenotypes to the control plants when treated with a heat stress. It is quite likely that those metabolites that were enhanced in their production do not necessarily have significant roles in combating heat stress. According to Wang et al. (2018), HMGR functions more efficiently at 28°C and radically declines when temperature rise to 40°C plus it is more active in young growing roots tip and apical buds than the matured plant tissues, showing a spatial regulation in its function. Although this is speculative, this might possibly be the reason for early senescence of older leaves and susceptibility of the *HMGR* transgenic plants to heat stress. Our initial idea assumed that *HMGR*-OE improves both plant tolerance to heat and accumulation of specialized metabolites and we could only confirm the latter in the *HMGR* transgenic lines (Figures 7, 8).

In conclusion, the *ER-*OE alleviates damaging effects caused by high temperature stress, imparting the thermo-tolerance in *P. ternata*. The mechanisms of how the *ER* gene controls cellular and molecular heat resistance is still unclear, needing more in-depth investigations to better understand how it governs heat stress shown here. Our study could not conclude on *HMGR*-OE regulated heat tolerance in *P. ternata* but the effect of this gene at the metabolite level was confirmed. This study has conclusively contributed toward generating heat-stress tolerant new *Pinellia* varieties with enhanced metabolite content. The *Pinellia* summer dormancy, which is intrinsic to the growth cycle of the species, can be altered through the introduction of a single heterologous gene. This novel finding provides a new genetic engineering strategy in *P. ternata* for future market needs.

## Data Availability Statement

The raw data supporting the conclusions of this article will be made available by the authors, without undue reservation, to any qualified researcher.

## Author Contributions

SJ, ZG, WC, and XH conceived and designed the experiments. SJ, ZG, and HY performed the experiments. SJ, XL, SH, and XH analyzed data. SJ, HY, and WC contributed in metabolite analysis. SJ, NM, XL, and XH wrote the manuscript. All authors checked the manuscript.

## Conflict of Interest

The authors declare that the research was conducted in the absence of any commercial or financial relationships that could be construed as a potential conflict of interest.

## References

[B1] Alexieva V. Sergiev I. Mapelli S. Karanov E. (2001). The effect of drought and ultraviolet radiation on growth and stress markers in pea and wheat. *PlantCell Environ.* 24 1337–1344. 10.1046/j.1365-3040.2001.00778.x

[B2] Balachowski J. A. Bristiel P. M. Volaire F. A. (2016). Summer dormancy, drought survival and functional resource acquisition strategies in California perennial grasses. *Ann. Bot.* 118 357–368. 10.1093/aob/mcw109 27325898 PMC4970370

[B3] Bansal S. Narnoliya L. K. Mishra B. Chandra M. Yadav R. K. Sangwan N. S. (2018). HMG-CoA reductase from Camphor Tulsi (*Ocimum kilimandscharicum*) regulated MVA dependent biosynthesis of diverse terpenoids in homologous and heterologous plant systems. *Sci. Rep.* 8:3547. 10.1038/s41598-017-17153-z 29476116 PMC5824918

[B4] Beauchamp C. Fridovich I. (1971). Superoxide dismutases: improved assays and an assay predictable to acrylamide gels. *Ann. Clin. Biochem.* 44 276–287. 10.1016/0003-2697(71)90370-8 4943714

[B5] Dai Z. Cu I. G. Zhou S.-F. Zhang X. Huang L. (2011). Cloning and characterization of a novel 3-hydroxy-3-methylglutaryl coenzyme A reductase gene from *Salvia miltiorrhiza* involved in diterpenoid tanshinone accumulation. *J. Plant Physiol.* 168 148–157. 10.1016/j.jplph.2010.06.008 20637524

[B6] Djanaguiraman M. Prasad P. V. Seppanen M. (2010). Selenium protects sorghum leaves from oxidative damage under high temperature stress by enhancing antioxidant defense system. *Plant Physiol. Biochem.* 48 999–1007. 10.1016/j.plaphy.2010.09.009 20951054

[B7] Froger A. Hall J. E. (2007). Transformation of plasmid DNA into *E. coli* using the heat shock method. *J. Vis. Exp.* 2007:253. 10.3791/253 18997900 PMC2557105

[B8] Fu C. (2005). The first prescription for the treatment of insomnia-the applications of banxia shumi decoction. *Jiangxi J. Tradit. Chin. Med.* 36 56–57.

[B9] Gillespie L. M. Volaire F. A. (2017). Are winter and summer dormancy symmetrical seasonal adaptive strategies? The case of temperate herbaceous perennials. *Ann. Bot.* 119 311–323. 10.1093/aob/mcw264 28087658 PMC5314652

[B10] Hasanuzzaman M. Nahar K. Alam M. M. Roychowdhury R. Fujita M. (2013). Physiological, biochemical, and molecular mechanisms of heat stress tolerance in plants. *Int. J. Mol. Sci.* 14 9643–9684. 10.3390/ijms14059643 23644891 PMC3676804

[B11] Hector V. Anne-Claire Morine D. Shpak E. Khodakovskaya M. V. (2012). Modification of tomato growth by expression of truncated ERECTA protein from *Arabidopsis thaliana*. *J. Exp. Bot.* 68 6493–6504. 10.1093/jxb/ers305 23096000

[B12] Hongying W. Shen W. Hongda L. Zhang L. Huang S. (2015). Study on the anti-motion sickness action of volatile oil constituents in *Pinellia ternate*. *Biomed. Res.* 26 230–234.

[B13] Hu S. (1989). Textual research of Chinese materia medica of *Pinellia ternata*. *Chin J. Chin. Mater. Med.* 149 6–9. 2695108

[B14] Iwasa M. Iwasaki T. Ono T. Miyazawa M. (2014). Chemical composition and major odor-active compounds of essential oil from PINELLIA TUBER (dried rhizome of *Pinellia ternata*) as crude drug. *J. Oleo Sci.* 63 127–135. 10.5650/jos.ess13092 24500103

[B15] Jin B. Jiang F. Yu M. Chen N. Ding Z. (2009). Agrobacterium tumefaciens mediated Chitinase and β-1,3-glucanase gene transformation for *Pinellia ternata*. *Chin. J. Chin. Materi. Med.* 34 1765–1767. 19894502

[B16] Kim Y. J. Lee O. R. Oh J. Y. Jang M.-G. Yang D.-C. (2014). Functional analysis of 3-Hydroxy-3-Methylglutaryl coenzyme a reductase encoding genes in triterpene saponin-producing ginseng. *Plant Physiol.* 165 373–387. 10.1104/pp.113.222596 24569845 PMC4012596

[B17] Kotak S. Larkindale J. Lee U. von Koskull-Döring P. Vierling E. Scharf K.-D. (2007). Complexity of the heat stress response in plants. *Curr. Opin. Plant Biol.* 10 310–316. 10.1016/j.pbi.2007.04.011 17482504

[B18] Krohn R. I. (2001). The colorimetric detection and quantitation of total protein. *Curr. Protoc. Cell. Biol.* 15, 1–28. 18228396 10.1002/0471143030.cba03hs15

[B19] Kumar D. Yusuf M. Singh P. Sardar M. Sarin N. (2014). Histochemical detection of superoxide and H2O2 accumulation in *Brassica juncea* seedlings. *Bio Protocol* 4:e1108 10.21769/bioprotoc.1108

[B20] Lee P. N. H. Lee W. Kim E. H. Jin Y. H. Seo E. K. Honhg J. (2016). Comprehensive chemical profiling of Pinellia species tuber and processed Pinellia tuber by gas chromatography - mass spectrometry and liquid chromatography - atmospheric pressure chemical ionization - tandem mass spectrometry. *J. Chromatogr. A.* 4 164–177. 10.1016/j.chroma.2016.10.033 27769531

[B21] Linzhou H. Huang T. Yasir A. Phillips A. L. Hu Y.-G. (2013). Isolation and characterization of *ERECTA* genes and their expression patterns in common wheat (*Triticum aestivum* L.). *J. Crop Sci.* 7 381–390.

[B22] Livak S. T. (2001). Analysis of relative gene expression data using real-time quantitative PCR and the 2(-Delta Delta C(T)) Method. *Methods* 25 402–408. 10.1006/meth.2001.1262 11846609

[B23] Loguercio L. L. Scott H. C. Trolinder N. L. Wilkins T. A. (1999). Hmg-coA reductase gene family in cotton (*Gossypium hirsutum* L.): unique structural features and differential expression of hmg2 potentially associated with synthesis of specific isoprenoids in developing embryos. *Plant Cell Physiol.* 40 750–761. 10.1093/oxfordjournals.pcp.a029602 10501034

[B24] Long S. P. Ort D. R. (2010). More than taking the heat: crops and global chang. *Curr. Opin. Plant Biol.* 13 241–248. 20494611 10.1016/j.pbi.2010.04.008

[B25] Ma X.-J. Li X.-W. Du J. Xue M. Li T. Luo Y.-C. (2006). Study on marking method for germplasm evaluation of *Pinellia ternata*. *Chin. J. Chin. Mater. Med.* 31 975–977. 17048641

[B26] Mahmoud S. S. Croteau R. B. (2002). Strategies for transgenic manipulation of monoterpene biosynthesis in plants. *Trends Plant Sci.* 7 366–373. 10.1016/S1360-1385(02)02303-8 12167332

[B27] Masle J. Gilmore S. R. Farquhar G. D. (2005). The *ERECTA* gene regulates plant transpiration efficiency in *Arabidopsis*. *Nature* 436 866–870. 10.1038/nature03835 16007076

[B28] Murashige T. Skoog F. (1962). A revised medium for rapid growth and bio assays with tobacco tissue cultures. *Physiol. Plant.* 15 473–497. 10.1111/j.1399-3054.1962.tb08052.x

[B29] Qu X. Zhao Z. Tian Z. (2017). ERECTA regulates cell elongation by activating auxin biosynthesis in *Arabidopsis thaliana*. *Front. Plant Sci.* 8:1688. 10.3389/fpls.2017.01688 29021806 PMC5623719

[B30] Shane M. W. McCully M. E. Canny M. J. Pate J. S. Huang C. Ngo H. L. (2010). Seasonal water relations of *Lyginia barbata* (Southern rush) in relation to root xylem development and summer dormancy of root apices. *New Phytol.* 185 1025–1037. 10.1111/j.1469-8137.2009.03143.x 20085620

[B31] Shen H. Zhong X. Zhao F. Wang Y. Yan B. Li Q. (2015). Overexpression of receptor-like kinase ERECTA improves thermotolerance in rice and tomato. *Nat. Biotechnol.* 33 996–1006. 10.1038/nbt.3321 26280413

[B32] Shiu S.-H. Karlowski W. M. Pan R. Tzeng Y.-H. Mayer K. F. X. Li W.-H. (2004). Comparative Analysis of the receptor-like kinase family in *Arabidopsis* and rice. *Plant Cell* 16 1220–1234. 10.1105/tpc.020834 15105442 PMC423211

[B33] Shpak E. Berthiaume C. Hill E. Torii K. U. (2004). Synergistic interaction of three ERECTA-family receptor-like kinases controls *Arabidopsis* organ growth and flower development by promoting cell proliferation. *Development* 13 149–151. 10.1242/dev.01028 14985254

[B34] Shpak E. D. (2013). Diverse roles of ERECTA family genes in plant development. *J. Integr. Plant Biol.* 55 1238–1250. 10.1111/jipb.12108 24016315

[B35] Song W. Y. Sun C. Wang Y. Peng Y. He C. (2012). Effects of pyrene on antioxidant systems and lipid peroxidation level in mangrove plants, Bruguiera gymnorrhiza. *Ecotoxicology* 21 1625–1632. 10.1007/s10646-012-0945-9 22678554

[B36] Tang J. Chen M. Liu W. (2008). Obtaining of anti-glyphosate Pinelliaternata and establishment of transformation system using glyphosate as selectable marker. *Tradit. Herbal Drugs* 39:585.

[B37] Tholl D. (2006). Terpene synthases and the regulation, diversity and biological roles of terpene metabolism. *Curr. Opin. Plant Biol.* 9 297–304. 10.1016/j.pbi.2006.03.014 16600670

[B38] Wang J. Y. Wang X.-D. Ma Y.-D. Fu L.-C. Zhou H.-H. Wand B. (2018). Physiological and ecological responses to drought and heat stresses in *Osmanthus fragrans* ‘Boyejingui’. *Chin. J. Plant Ecol.* 42 681–691. 10.17521/cjpe.2018.0017

[B39] Wang R. Ni J. Ma R. (1995). Volatile oils of *Pinellia ternata*. *Chin. Pharm. J.* 30 457–459.

[B40] Wang X. S. Wu Y. F. Ma J. Y. Qilu Q. L. S. (2008). Study on chemical components and pharmacological activities of *Pinellia ternata*. *Pharm. Aff.* 27 101–103.

[B41] Wu X.-Y. Zhao J.-L. Zhang M. Li F. Zhao T. Yang L.-Q. (2011). Sedative, hypnotic and anticonvulsant activities of the ethanol fraction from rhizoma pinelliae praeparatum. *J. Ethnopharmacol.* 135 325–329. 10.1016/j.jep.2011.03.016 21402138

[B42] Xing H. Peng G. Xin L. Wei L. (2011). PdERECTA, a leucine-rich repeat receptor-like kinase of poplar, confers enhanced water use efficiency in *Arabidopsis*. *Planta* 234 229–241. 10.1007/s00425-011-1389-9 21399949

[B43] Xu Q. Xu X. Shi Y. Xu J. Huang B. (2014). Transgenic tobacco plants overexpressing a grass PpEXP1 gene exhibit enhanced tolerance to heat stress. *PLoS One* 9:e100792. 10.1371/journal.pone.0100792 25003197 PMC4086929

[B44] Xu T. Zhang L. Sun X. Tang K. (2005). Efficient in vitro plant regeneration of *Pinellia ternata* (Thunb) Breit. *Acta Biol. Crac. Ser. Bot.* 2 27–32.

[B45] Yang Y. Li R. Qi M. (2000). *In vivo* analysis of plant promoters and transcription factors by Agroinfiltration of tobacco leaves. *Plant J.* 22 543–551. 10.1046/j.1365-313x.2000.00760.x 10886774

[B46] Zang X. Geng X. Wang F. Liu Z. Zhang L. Zhao Y. (2017). Overexpression of wheat ferritin gene TaFER-5B enhances tolerance to heat stress and other abiotic stresses associated with the ROS scavenging. *BMC Plant Biol.* 17:14. 10.1186/s12870-016-0958-2 28088182 PMC5237568

[B47] Zhang J. Y. Guo Q. S. Zheng D. S. (2013). Genetic diversity analysis of *Pinellia ternata* based on SRAP and TRAP markers. *Biochem. Syst. Ecol.* 50 258–265. 10.1016/j.bse.2013.03.052

[B48] Zhang K. W. Wu H. Wu L. L. (2002). A study on component of aliphatic acid in Rhizoma *Pinelliae ternata* Praeparata. *J. Nanjing TCM Univ.* 18 291–302.

[B49] Zhu Y. Zhu G. Guo Q. Zhu Z. Wang C. Liu Z. (2018). Cloning and expression of a new cytoplasmic small heat shock protein gene from *Pinellia ternata*. *Acta Physiol. Plant.* 40 1–44. 10.1007/s11738-018-2605-z

[B50] Zuo Z. Fan H. Wang X. Zhou W. Li L. (2012). Purification and characterization of a novel plant lectin from Pinellia ternata with antineoplastic activity. *SpringerPlus* 1:13. 10.1186/2193-1801-1-13 23961344 PMC3725870

