# Supplementary material for: Breaking the Summer Dormancy of Pinellia ternata by Introducing a Heat Tolerance Receptor-Like Kinase ERECTA Gene
Source: Front Plant Sci. 2020 Jun 24;11:780. doi: 10.3389/fpls.2020.00780 (PMC7326942; doi:10.3389/fpls.2020.00780)
Supplement: Supplementary file 1 [file Data_Sheet_1.PDF]

# Supplemetary Materials

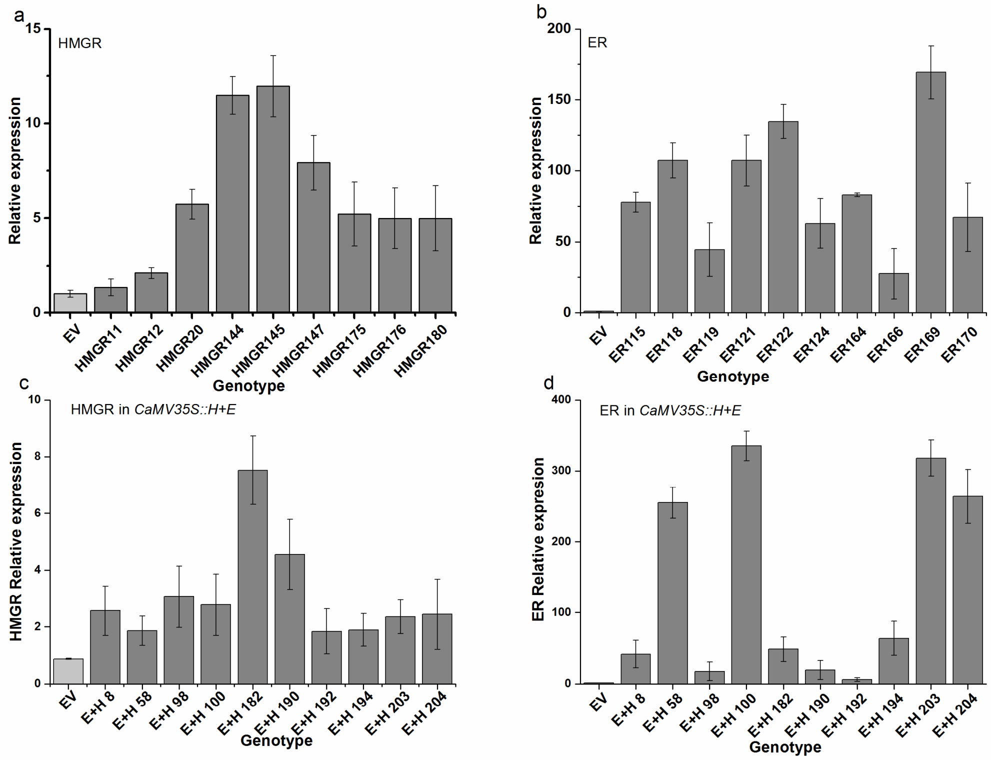


**Figure S1** The real time RT-PCR transcript levels in primarily screening of the transgenic lines. The relative expression levels of (A) HMGR, (B) ER and (C) HMGR and (D) ER in H+E in 10-week-old *P. ternata* transgenic lines overexpressing *HMGR, ER* or *H+E* under the *CaMV35S* promoter. The *Actin* gene was used as a control to normalize internal expression level.

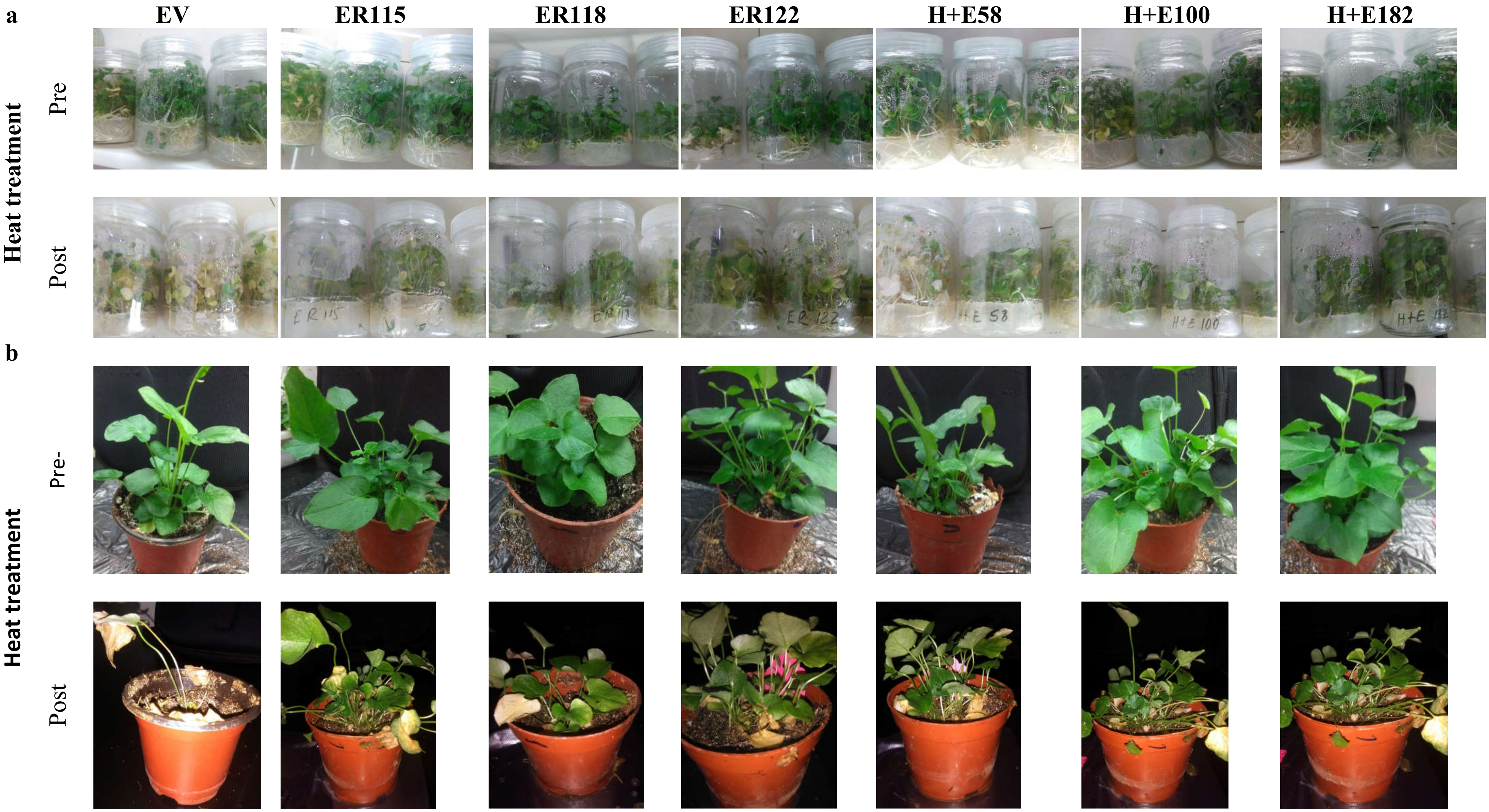


**Figure S2** Preliminary heat treatment of *P. ternata* seedlings for selection of thermo-tolerant lines. (A) seven-week-old seedling grown on MS medium (n=10), (B) Ten-week-old seedlings transplanted to soil. The seedlings were treated with a high temperature (40$^{o}$C, 7-d) (n=20).

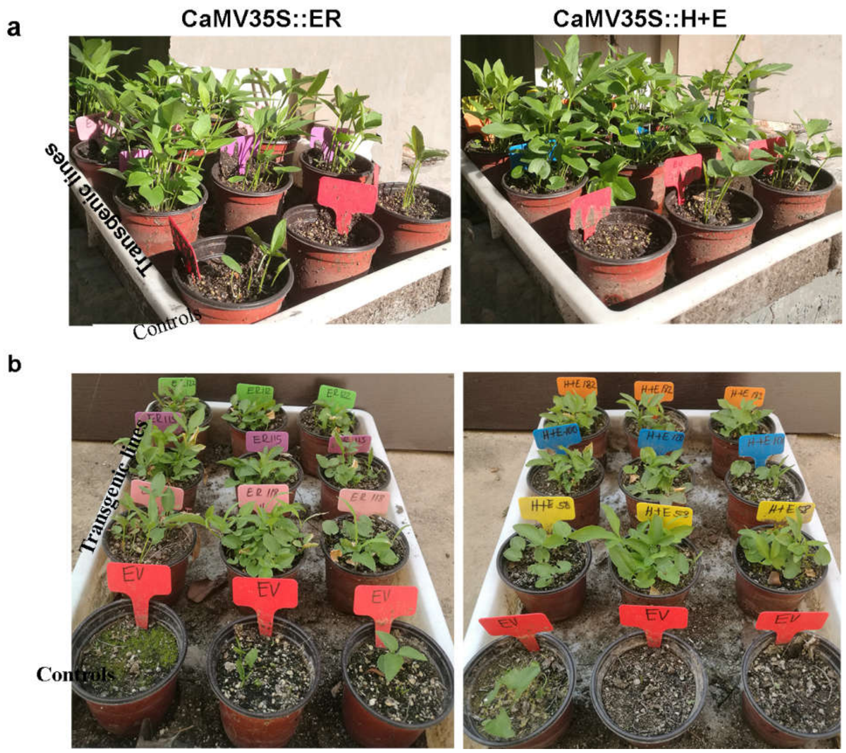


**Figure S3** Pictures of transgenic *Pinellia* plants during open field trials. The 16-week-old plants that grown during spring (A) and summer (B) seasons. The seedlings were grown in a growth chamber for 8 weeks and acclimatized in growth culture room for 2 weeks then transferred to open natural field. The study was conducted for two consecutive year, 2018 and 2019 (n=30, for each season)

a

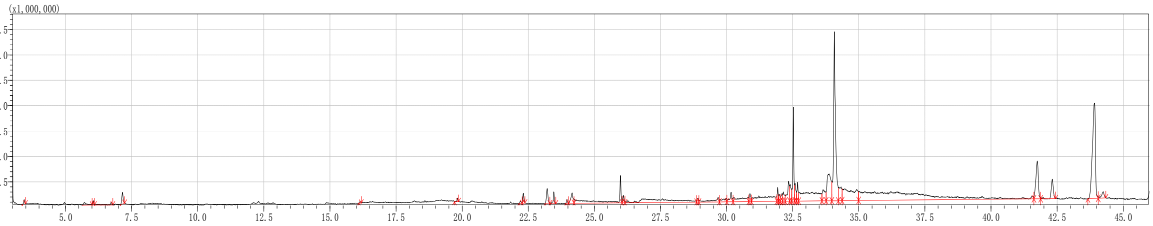
(x1,000,000)
3.5
3.0
2.5
2.0
1.5
1.0
0.5
5.0 7.5 10.0 12.5 15.0 17.5 20.0 22.5 25.0 27.5 30.0 32.5 35.0 37.5 40.0 42.5 45.0

b

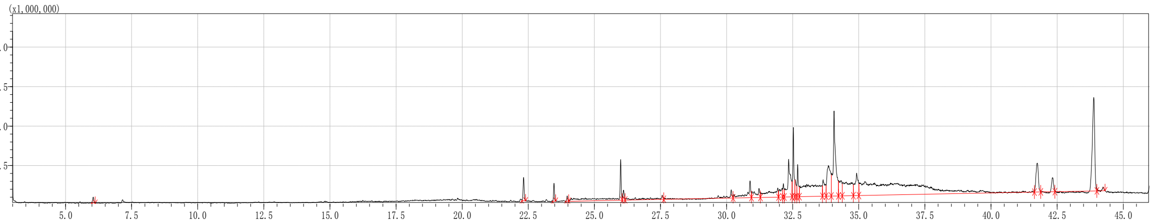
(x1,000,000)
2.0
1.5
1.0
0.5
5.0 7.5 10.0 12.5 15.0 17.5 20.0 22.5 25.0 27.5 30.0 32.5 35.0 37.5 40.0 42.5 45.0

c

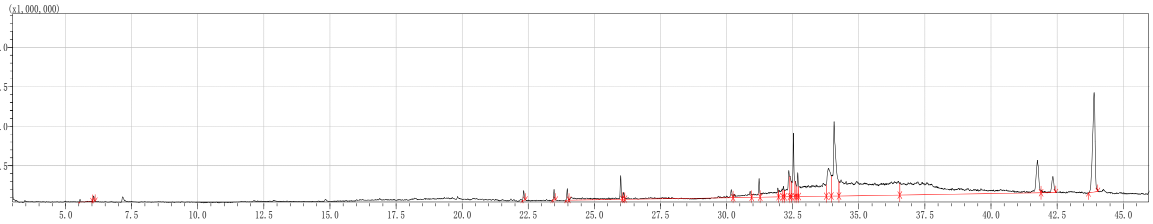
(x1,000,000)
2.0
1.5
1.0
0.5
5.0 7.5 10.0 12.5 15.0 17.5 20.0 22.5 25.0 27.5 30.0 32.5 35.0 37.5 40.0 42.5 45.0

d

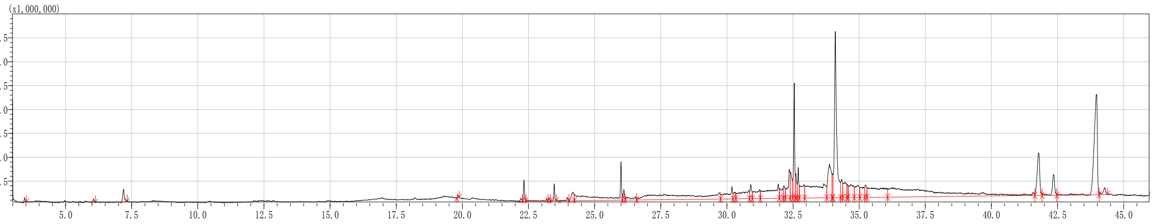
(x1,000,000)
3.5
3.0
2.5
2.0
1.5
1.0
0.5
5.0 7.5 10.0 12.5 15.0 17.5 20.0 22.5 25.0 27.5 30.0 32.5 35.0 37.5 40.0 42.5 45.0

e

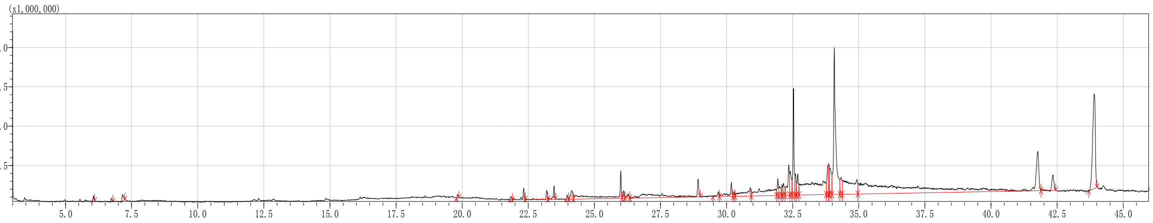
(x1,000,000)
2.0
1.5
1.0
0.5
5.0 7.5 10.0 12.5 15.0 17.5 20.0 22.5 25.0 27.5 30.0 32.5 35.0 37.5 40.0 42.5 45.0

f

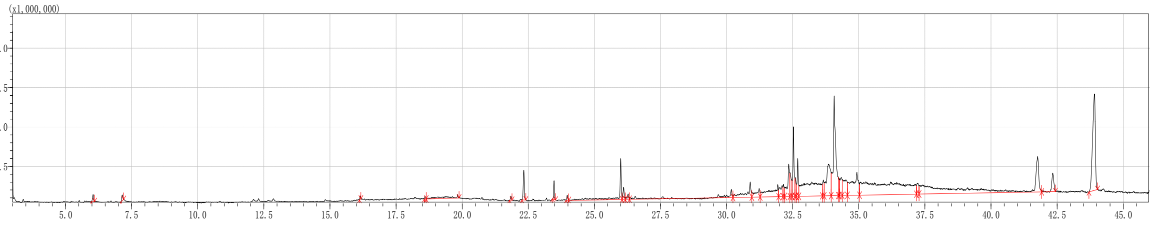
(x1,000,000)
2.0
1.5
1.0
0.5
5.0 7.5 10.0 12.5 15.0 17.5 20.0 22.5 25.0 27.5 30.0 32.5 35.0 37.5 40.0 42.5 45.0

g

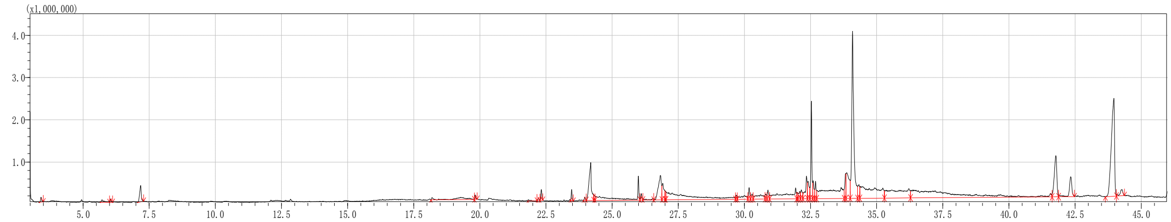


h

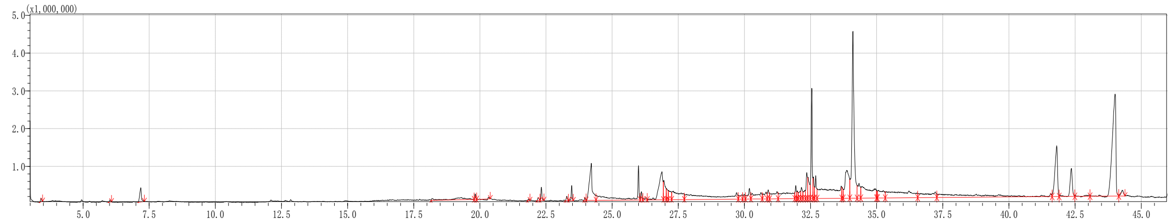


i

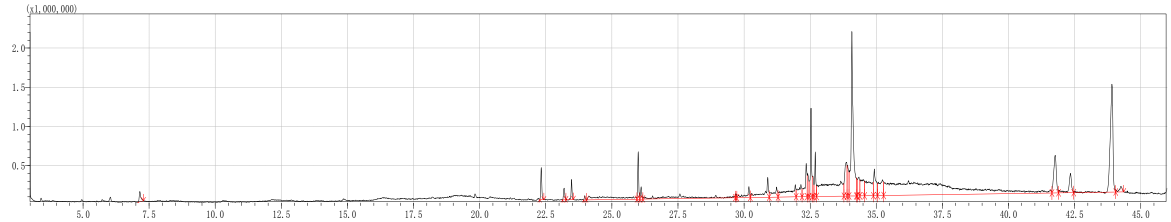


j

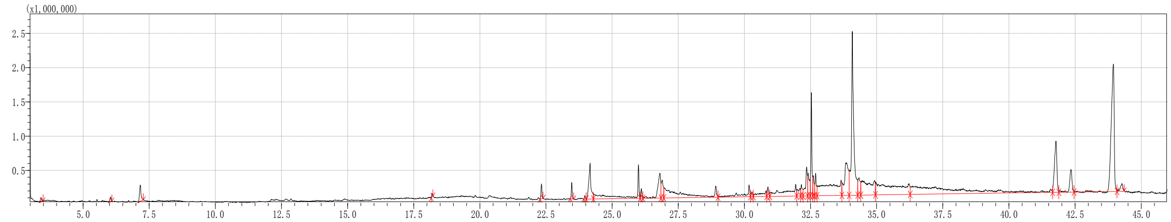


k

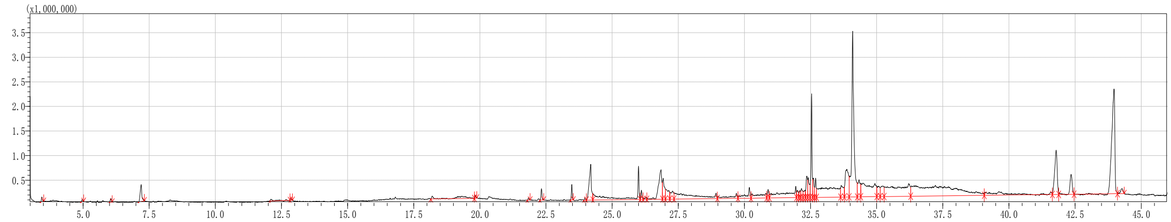


l

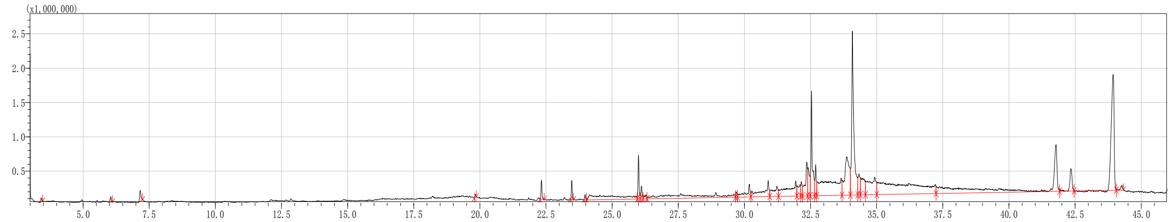


**Figure S4** Total ion chromatograms of the control and transgenic *P. ternata* methanolic tuber extracts. Scanning was performed from 60 to 665 atomic mass units and the identified individual compound is displayed together with its retention time in Table S6. (A-C) *EV*, (D-F) *ER*, (G-I) *HMGR* and (J-L) H+E genotypes

**Table S1** Primers used for amplifying genes and quantifying gene expression

| Gene | Primers | Sequences (5'-3') |
| --- | --- | --- |
| Releasing *HMGR* from a vector | HMGR forward | CGGGGGATCCTCATTGTTGGCTTCTTAAATCTTGT |
| | HMGR reverse | GCTTATCGATTTAAGACTGTGTGTGAGAAAGTTCC |
| *HMGR* cloning | *Nco*l-HMGR forward | TTGACCATGGTACAAAGTTTAGATAAGAATTTCC |
| | *BstE*II-HMGR reverse | GAGCTGGTCACCTCATTGTTGGCTTCTTAAAT TTG |
| *pCA1301-HMGR-ER* construction | | |
| NcoI and BSTEII cleavage sites | *Nco*I-HMGR forward | TTGACCATGGTACAAAGTTTAGATAAGAATTTCC |
| | *delBs*tII reverse | GTTGGTAGCCACCACCACGCGCTT |
| BSTEII cleavage site and part of NcoI cleavage site | *delBs*tII forward | GTGGTGGTGGCTACCAACGTATAGCT |
| | BSTEII-HMGR reverse | GAGCTGGTCACCTCATTGTTGGCTTCTTAAATCTTG |
| NcoI, BSTEII cleavage site on HMGR fragment | NcoI-HMGR forward | TTGACCATGGTACAAAGTTTAGATAAGAATTTCC |
| | BSTEII-HMGR reverse | GAGCTGGTCACCTCATTGTTGGCTTCTTAAATCTTG |
| Recombinant plasmids identification | Forward | TTGACCATGGTACAAAGTTTAGATAAGAATTTCC |
| | Reverse | GAGCTGGTCACCTCATTGTTGGCTTCTTAAATCTTG |
| RT-qPCR | | |
| *HMGR* | Forward | GTTATGAATGGCATACATGCCG |
| | Reverse | GTCATAGGCACTTCTATTGTACC |
| *ER* | Forward | CCCCGAGCATTTCAAAAGCT |
| | Reverse | TGCTCCAAATCACCAAGGGA |
| *Actin* | Forward | GGTATTGGGCTGGGTTTTGG |
| | Reverse | AGATATCACCTCAGCCGCTG |

**Table S2** The phenotypic measures of the *HMGR* transgenic lines challenged with high temperatures

| **Genotype** | **Chlorophyll content** | | | | **Surviving and recovering rate (40ºC, 15-d)** | | | | | | | | **Long-term (35ºC, 90-d)** |
|---|---|---|---|---|---|---|---|---|---|---|---|---|---|
| | 0 h | 24 h | 24 h | 72 h | 0-d | 1-d | 3-d | 5-d | 10-d | 15-d (Rd-1) | 7-d | 15-d | |
| EV | 47.08±0.93 | 41.45±1.14 | 35.19±0.48 | 11.28±0.71 | 100 | 65.03±4.9 | 34.68±3.02 | 38.56±3.5 | 39.61±3.35 | 36.08±3.93 | 31.28±2.62 | 52.03±4.05 | 12.63±0.92 |
| HMGR144 | 46.38±0.79 | 40.87±0.98 | 33.24±0.48 | 13.35±0.73 | 100 | 64.71±3.3 | 28.23±3.4 | 33.14±4 | 34.68±3.4 | 31.44±3.9 | 29.89±2.41 | 52.17±4.66 | 13.65±1.95 |
| HMGR145 | 45.58±0.93 | 40.16±1.19 | 33.79±0.47 | 12.49±0.69 | 100 | 66.65±4.2 | 18.26±2.62 | 23.86±2.85 | 22.87±1.91 | 24.80±2.68 | 29.12±2.05 | 50.37±3.97 | 15.27±3.47 |
| HMGR147 | 47.49±0.92 | 42.25±1.19 | 34.60±0.48 | 12.50±0.75 | 100 | 63.61±3.9 | 12.37±2.74 | 16.91±2.99 | 17.41±2.05 | 18.21±2.59 | 30.74±2.33 | 54.17±4.86 | 13.42±1.66 |

*EV,* empty vector*; HMGR,* 3-hydroxy-3-methyl glutaryl coenzyme A reductase; d, day; Rd, recovery days

**Table S3** Physio-biochemical assays for *HMGR* transgenic lines treated with a high temperature (40$^{o}$C-15-d)

| Genotype | Relative water content | | | Ion leakage | | | | Lipid peroxidation | | $H_2O_2$ concentration | | Total protein content | | SOD activity | |
|---|---|---|---|---|---|---|---|---|---|---|---|---|---|---|---|
| | 0 h | 24 h | 48 h | 0 h | 24 h | 4 8h | 72 h | 0 h | 48 h | 0 h | 72 h | 0 h | 48 h | 0 h | 48 h |
| EV | 95.34± 0.4 | 87.58± 1 | 87.33± 0.84 | 14.8± 0.92 | 16.42± 1.4 | 21.6± 4.1 | 31.6± 4.5 | 0.25± 0.07 | 0.44± 0.11 | 3.26± 0.16 | 5.73± 1.34 | 1.38± 0.11 | 0.610± 0.12 | 0.07± 0.01 | 0.18± 0.01 |
| HMGR144 | 97.42± 0.29 | 87.34± 1.47 | 86.35± 1.4 | 14.8± 1.6 | 17.90± 1.4 | 22.2± 5.4 | 35.4± 4.6 | 0.21± 0.05 | 0.28± 0.08 | 3.45± 0.33 | 5.29± 0.24 | 1.64± 0.13 | 0.650± 0.08 | 0.07± 0.011 | 0.13± 0.06 |
| HMGR145 | 96.82± 0.5 | 87.47± 1.6 | 83.71± 1.3 | 14.2± 1.3 | 18.70± 1.3 | 23.2± 3.4 | 36.4± 4.25 | 0.26± 0.02 | 0.26± 0.02 | 3.41± 0.15 | 5.07± 1.03 | 1.90± 0.26 | 0.710± 0.05 | 0.06± 0.01 | 0.11± 0.06 |
| HMGR147 | 96.18± 1.9 | 86.19± 1.4 | 85.35± 1.02 | 15.4± 1.7 | 18.70± 1.4 | 23.2± 3.5 | 36.4± 4.4 | 0.29± 0.03 | 0.29± 0.03 | 3.06± 0.21 | 4.59± 0.41 | 1.80± 0.09 | 0.710± 0.06 | 0.08± 0.01 | 0.12± 0.06 |

*EV,* empty vector*; HMGR,* 3-hydroxy-3-methyl glutaryl coenzyme A reductase

**Table S4** Monthly average temperatures, humidity and rainfall in Wuhan, China, 2018 & 2019

| | | Jan | Feb | Mar | Apr | May | Jun | Jul | Aug | Sep | Oct | Nov | Dec |
|---|---|---|---|---|---|---|---|---|---|---|---|---|---|
| Temperature | | | | | | | | | | | | | |
| 2018 | Max | 6 | 11 | 18 | 24 | 28 | 31 | 34 | 34 | 29 | 25 | 19 | 10 |
| | Ave | 4 | 8 | 14 | 20 | 24 | 28 | 31 | 31 | 25 | 23 | 17 | 8 |
| | Min | 1 | 5 | 11 | 15 | 21 | 24 | 27 | 27 | 22 | 19 | 14 | 6 |
| 2019 | Max | 10 | 9 | 20 | 26 | 29 | 32 | 34 | 37 | 31 | 27 | 18 | 11 |
| | Ave | 8 | 7 | 17 | 23 | 27 | 30 | 32 | 34 | 28 | 24 | 16 | 9 |
| | Min | 5 | 5 | 12 | 1 | 21 | 24 | 27 | 28 | 22 | 18 | 13 | 8 |
| Humidity | 2018 | 71 | 57 | 67 | 63 | 65 | 63 | 67 | 65 | 62 | 45 | 59 | 62 |
| | 2019 | 63 | 72 | 57 | 60 | 52 | 64 | 66 | 60 | 53 | 49 | 76 | 66 |
| Rainfall | 2018 | 48.74 | 21.9 | 64.85 | 58.77 | 84.27 | 59.14 | 90.32 | 49.86 | 30.04 | 17.9 | 87.7 | 95.6 |
| | 2019 | 60.6 | 77 | 134.3 | 113.3 | 117.8 | 142.5 | 136.4 | 74.4 | 21.3 | 8.6 | 52 | 91.2 |

**Table S5** Plant growth performance evaluation during spring season

| Genotype | Root length (cm) | Fresh biomass (g) | Dry biomass (g) | Tuber fresh wt (g) | Tuber dry wt (g) |
|---|---|---|---|---|---|
| EV | 12.21±1.26 | 8.27±0.91 | 1.85±0.41 | 5.23±0.89 | 3.95±1.36 |
| ER115 | 12.04±1.11 | 8.88±0.69 | 1.88±0.57 | 6.69±1.21 | 6.05±1.14 |
| ER118 | 12±1.26 | 10.97±0.69 | 2.71±0.35 | 4.82±0.82 | 3.66±0.94 |
| ER122 | 12.42±1.6 | 10.52±0.87 | 2.53±0.66 | 6.91±2.35 | 5.78±0.78 |
| H+E58 | 12.05±1.22 | 9.38±0.94 | 2.13±0.35 | 7.81±2.17 | 6.1±0.53 |
| H+E100 | 12.13±1.11 | 10.54±0.79 | 2.22±0.41 | 7.78±1.75 | 6.08±0.36 |
| H+E182 | 12.03±1.53 | 9.95±0.91 | 2.21±0.65 | 8.72±1.72 | 6.22±0.79 |

**Table S6** Relative intensity of abundantly detected compounds in controls and *HMGR* overexpressing transgenic lines

| No. | RT | Name of the compound | M.F | M.W ($gmol^{-1}$) | Peak area (%) | | | |
|---|---|---|---|---|---|---|---|---|
| | | | | | Genotype | | | |
| | | | | | ***EV*** | ***ER*** | ***HMGR*** | ***H+E*** |
| 1 | 3.44 | Aceturic acid, 2TMS derivative | $C_7H_{15}NO_3Si$ | 189 | 0.4±0.12 | ND | 0.4±0.1 | 0.33±0.02 |
| 2 | 22.22 | 3-(Prop-2-enoyloxy) dodecane | $C_{15}H_{28}O_2$ | 240 | 1.6±0.1 | ND | 0.3±0.12 | Tr |
| 3 | 22.33 | 2-Hydroxy-4,6-dimethylbenzaldehyde | $C_9H_{10}O_2$ | 150 | 1±0.5 | 1.5±0.7 | 0.63±0.2 | 1.1±0.4 |
| 4 | 23.97 | Benzoic acid, 3-formyl- | $C_8H_6O_3$ | 150 | 1.17±0.5 | 0.33±0.1 | 0.9±0.12 | ND |
| 5 | 24.12 | Desulphosinigrin | $C_{10}H_{17}NO_6S$ | 279 | 0.3±0.2 | 0.4±0.04 | 5.9±0.1 | 4±0.8 |
| 6 | 26.0 | 10-Methyl-E-11-tridecen-1-ol propionate | $C_{17}H_{32}O_2$ | 268 | ND | 2.1±0.2 | 0.4±0.08 | 2.4±0.25 |
| 7 | 26.11 | 7-Hexadecenoic acid, methyl ester (Z)- | $C_{17}H_{32}O_2$ | 268 | 0.94±0.1 | 1.1±1.03 | 3.23±0.9 | 3±1.2 |
| 8 | 27.28 | 1-β -d-Ribofuranosyl-3-[5-tetraazolyl]-1,2,4-triazole | $C_8H_{11}N_7O_4$ | 269 | 1.23±0.3 | Tr | 0.4±0.1 | 0.11±0.03 |
| 9 | 30.18 | Ethylene brassylate | $C_{15}H_{26}O_4$ | 270 | 0.8±0.07 | Tr | 0.66±0.01 | 0.6±0.05 |
| 10 | 32.16 | 1,3,5-Trisilacyclohexane | $C_3H_{12}Si_3$ | 132 | Tr | 0.32±0.12 | 0.3±0.02 | 0.84±0.44 |
| 11 | 32.53 | 9,9-Dimethoxybicyclo[3.3.1]nona-2,4-dione | $C_{11}H_{16}O_4$ | 212 | 6.8±0.1 | 0.23±0.11 | 3.1±2.3 | 6.7±0.7 |
| 12 | 32.69 | Bis (2-ethylhexyl) phthalate | $C_{24}H_{38}O_4$ | 390 | 1.2±0.8 | ND | 1.1±0.4 | 0.9±0.2 |
| 13 | 34.08 | 7-Hexadecyn-1-ol | $C_{16}H_{30}O$ | 238 | 17.12±3.8 | 23.7±6.3 | 19.9±0.13 | 21±1.2 |
| 14 | 41.75 | 4,8,13-Cyclotetradecatriene-1,3-diol, 1,5,9-trimethyl-12-(1-methylethyl)- | $C_{20}H_{34}O_2$ | 306 | 3.6±0.3 | 2.8±1.1 | 8±0.5 | 6.26±1.3 |
| 15 | 42.36 | Cholestan-3-ol, 2-methylene-, (3.β. 5.α )- | $C_{28}H_{48}O$ | 400 | 3.3±0.2 | 2.2±0.12 | 1.2±0.11 | Tr |
| 16 | 43.75 | (-)-Globulol | $C_{15}H_{26}O$ | 222 | 21.1±3.1 | 18.6±2.9 | 2.6±1.13 | 7.3±0.35 |
| 17 | 43.98 | Aromadendrene-4, 10-diol | $C_{15}H_{26}O_2$ | 238 | 8.7±1.6 | 7.3±0.1 | 24.3±0.74 | 23.5±1.7 |

The intensity of peak is the mean of three *HMGR* overexpressing genotypes or the mean of three control plants. RT, Retention time; M.F, Molecular formula; M.W, Molecular weight; Tr, trace $\leq$ 0.1; ND, not detected.
